# Evaluation of Changes in Polymer Material Properties Due to Aging in Different Environments

**DOI:** 10.3390/polym14091682

**Published:** 2022-04-21

**Authors:** Ivana Salopek Čubrić, Goran Čubrić, Ines Katić Križmančić, Monika Kovačević

**Affiliations:** 1 Department of Textile Design and Management, Faculty of Textile Technology, University of Zagreb, 10000 Zagreb, Croatia; ivana.salopek@ttf.unizg.hr; 2 Department of Clothing Technology, Faculty of Textile Technology, University of Zagreb, 10000 Zagreb, Croatia; 3 2K IDEJA, 10000 Zagreb, Croatia; ikatickri@ttf.unizg.hr; 4 Department of Chemistry and Biochemistry, Faculty of Food Technology and Biotechnology, University of Zagreb, 10000 Zagreb, Croatia; mkovacevic@pbf.hr

**Keywords:** aging, polymer, polyester, yarn, elongation, force, topography, structure, ATR-FTIR, moisture

## Abstract

With the increase in awareness of the importance of engagement in physical activities, high requirements have been placed on polymers intended for use in sports. A number of authors investigated the influence of aging factors on the performance of the polymer. Still, there is a lack of aging protocols that would be product-centered, especially when high performance is imperative. This paper presents a new approach to polymer aging and examines the change of the identified set of properties due to aging under different conditions, and the duration of each (topography, thickness, moisture management, elongation, and bursting force). The results of the testing revealed the increase in thickness due to exposure, especially to the sun-exposed materials. The ability of materials to elongate until the moment of rupture decreases due to exposure to the sun (strong relationship to the time of exposure; R^2^ reaches 0.99) and the bursting force (up to 6.8%). Furthermore, results indicate the significantly impaired capacity of the polymer material to absorb moisture. The results of measurements indicated (derived) by spectroscopic studies, based on the ATR-FTIR (attenuated total reflectance) method, showed that there was no detectable influence of aging in the sun or shade on the chemical structure of polyester samples.

## 1. Introduction

To study the changes that occur in polymers due to aging, specifically due to exposure to different weather conditions (outdoors or in the laboratory), it is necessary to understand and define all the parameters that affect aging. Previously published papers defined the list of potentially significant aging parameters, such as radiation, temperature, humidity, oxygen level, precipitation and condensed moisture, particulate matter, gaseous pollutants, and stress [1]. 

In the production of sportswear, the best known and most commonly used type of polyester is poly (ethylene terephthalate), i.e., PET. In terms of production volume and consumption, polyester fibers rank first in the world in the group of artificial fibers. Its elasticity, durability, ease of maintenance, and resistance to wear make it ideal for a number of applications [2]. PET is further characterized by good chemical resistance, especially to acids, excellent resistance to cleaning solvents, and bleaching agents [3]. Its recyclability is also highly valued in terms of ecological and sustainability issues. PET is relatively resistant to UV radiation, but prolonged exposure to the sun can lead to degradation, cleavage of macromolecular chains, and further destruction of the structure. The strength of standard types of PES fibers is in the range of 30–70 cN/tex [4]. A conducted experiment showed that the PET fibers have a lower tensile strength at break than standard PES fibers [5]. In terms of breaking elongation, this polymer is in the range of 20–30% [4].

With the increase in awareness of the importance of wellbeing and engagement in physical activities, high requirements have been placed on polymers and polymer structures intended for use in sports. Such materials are more exposed to extreme conditions than materials intended for everyday clothing, which causes a decrease in durability and degradation, consequently negatively affecting performance issues.

Different approaches have been proposed for the enhancement of the properties of polymers, as well as their durability when exposed to aging [6,7,8,9,10]. One of these approaches refers to the development of an upscalable melt-spinning method for the production of poly(hydroxybutyrate-co-3-hexanoate), i.e., PHBH [6]. The measurements revealed the positive impact of the method that resulted in increased tensile strength. Marjo et al. [8] used ATR-FTIR spectroscopy to confirm that the elastomeric component in the structure of polymer materials has a tendency to disintegrate over time due to inadequate humidity in the storage space. In previous studies, the effects of washing treatments (normal bleach, enzyme, enzyme bleach, super stone, enzyme perlit) on the performance of polymers were observed. According to the results, the decrease in the strength of polyester materials is up to 11.54%. When polymer materials are combined with natural or derivate fibers, the decrease is even higher, reaching 20.51% for the combination of polyester and viscose [11].

A number of authors have investigated the impact of aging factors on the performance of polymers. Andersen et al. studied the chemical and physical degradation of polyester and changes in the chemical composition of polymers. According to their study, polyesters subjected to real-time exposure (total of 2928 h) showed significant degradation of mechanical strength (the reduction ranged from 43% to 70%) [12]. The results also showed that PET-R, PBT, PETG, and PCTT polyesters became more brittle due to the aging process. In another study, scientists observed the change in water vapor resistance after exposure to different aging conditions at different seasons. The results indicated that after the exposure to external weather conditions, all specimens showed reduced resistance to water vapor. More specifically, the decrease after exposure during the hot season was 11.4%, and after exposure during the cold season was 16.7% [13]. The investigation of polymer-coated materials aging indicated a decrease in heat resistance [14]. Precisely, after three months of exposure to hot weather conditions, the decrease was 13%, and 25% after aging during the cold season. The obtained results also showed that the polyurethane coating protected and prevented the deterioration of the substrate [14]. In terms of structural parameters, the exposure of specimens to weathering affected the change of vertical density, which increased to 31%. It further affected a significant increase in mass per unit area to 26% [15]. In both horizontal and vertical directions of the structure, the breaking force was reduced up to 54%, and the heat resistance up to 18%.

The development of relevant protocols for material aging is an ever-going issue. Efforts have been made to develop the aging protocols for PES and PA used in aquatic media [16,17]. The authors reported a reduction in breaking force due to the conducting of aging of up to 40%. Furthermore, in the comparison of outdoor and indoor aging, the changes in the surface are confirmed to be more prominent after aging outdoors. A study of the impact of polymer aging in water (seawater vs. pool water) indicated an increase in the thickness of polymer aged in seawater by up to 10%. At the same time, the increase in thickness due to aging in pool water was barely notable. Numerous researchers have dealt with human physiology with an emphasis on the influence of sweat, temperature, and wear on the aging of polymers [18,19,20,21,22,23]. In order to draw attention to the importance of the influence of sweat on the properties of polymer structures, researchers provided guidelines for the design of sportswear and the selection components based on body mapping and body sweating patterns [18,19].

Infrared spectroscopy is an efficient and fast method often used to clarify and improve studies related to the textile industry, such as investigation of hydrolysis of PET fibers by FTIR-ATR [24], evaluating the fiber properties and the number of impurities [25], identifying thermal changes in the chemical structure of cotton fibers [26], identification of fibers [27] and forensic analysis of polyester fibers [28]. Considering the wide range of IR spectroscopy applications mentioned above, it is obvious that ATR-equipped FT-IR spectroscopy is a suitable method for analyzing the influence of different effects on the aging of the material. Changes in the chemical bonding of fibers can be caused by different external conditions depending on the type and vulnerability of chemical bonds [29]. The chemical stability of these materials is, on the one hand, a great concern related to the concourse of the textile with a view to its biodegradability [30], which is, on the other hand, a goal when fibers need to be resistant and of adequate strength. It is well known that exposure of different kinds of textile fibers to high or extreme temperatures leads to a different stage of degradation of material caused by changes in chemical bonding [26,31]. Polyester is a type of fiber that has ester bonds obtained by the reaction of different types of acids and alcohols, whose structure is responsible for the prerequisite material attribute. PET polyester is a notably chemical resistant material, thanks to an aromatic segment (terephthalate) coupled to a short aliphatic chain (ethylene), which causes rigidity of polymer and high thermal stability, with only a few degradation processes caused by humidity and UV light [29,32,33].

Despite the interest in the improvement of polymer structures, to our knowledge, scientists have not previously addressed the development of different aging protocols and investigation of polymer structures in the context of the use for athletes in team sports such as football. This paper presents a new approach to polymer aging with a defined aging protocol that corresponds to the usual training praxis, which is confirmed through a detailed preliminary investigation. The paper aims to examine the change of a carefully identified set of properties due to aging under different conditions and durations. More specifically, the focus is on polymer structure topography, thickness, moisture management, elongation at break, and bursting force. In light of the previously described importance of ATR-FTIR screening, we found that this method would be a good tool to deduce the influence of exposure to aging or chemical changes in the material, and so this method was used as well.

## 2. Methods

### 2.1. Aging Protocol

When defining the aging protocol, the establishment of the time required for an experiment may be in terms of adapting existing practices or selection of a duration time equivalent to the warranties offered to users. Still, the selected conditions of exposure should always simulate as closely as possible the conditions under which the polymer material is expected to perform.

In the absence of previously defined protocols (as reported in the Introduction), within the experimental part is initiated the development of aging polymer materials when used in professional football. The methods used to obtain insight into the use in real sports situations are direct semi-structured interviews, focus group research, online questionnaires with a combination of open- and closed-ended questions and literature review. Based on the outcomes of the previously defined protocols, the Polymer Aging Protocol for Football (PAPF) is precisely defined. The PAPF includes the following 6 main stages:

1. Selection of the target group;
2. Defining training conditions;
3. Selection of material samples;
4. Defining aging factors;
5. Defining the order of aging;
6. Selection of test methods.

With respect to the PAPF protocol, the aging conducted within the experimental part of this paper was focused on cadets as the target group. The cadets train three times a week for an hour, which is a total of 12 h per month, i.e., 720 min. Overall, during a 60-min long training, an athlete’s sportswear is approximately 45 min in contact with produced sweat, i.e., 540 min or 9 h per month. Workouts take place outdoors; the jerseys were also exposed to outdoor conditions such as sun and shade. Polymer materials from 100% PET were used for the experiment. The weft structures were created by interweaving polymer yarns to ensure elasticity and porosity of the material, allowing increased comfort and improved mass transfer through the structure. The description of the polymers (assigned as F1–F5), including the description of the structure, mass, and yarn fineness, can be found in Table 1.

The main aging factors are defined as exposure to weathering, continuous sweating, and care factors. Training conditions and aging factors based on 12 workouts (1 month) and 24 workouts (2 months) are shown in Table 2.

Polymer materials were exposed to in vivo weathering during the summer season (July–August 2021) at coordinates 45°48′55.4364″ N and 15°57’59.6448″ E. The average air temperature was 30.91 °C (ranging from 29 °C to 34 °C, with CV 5.53%). The UV index averaged 6 (it varied between 5 and 7, with CV at 6.9%). Humidity averaged 50% (i.e., it ranged from 49 to 56%, with CV 4.28%). The average wind speed was 7.9 km/h (7 km/h to 9 km/h, CV 7.8%), and the air pressure was 1010 mbar (varied from 1008 mbar to 1015 mbar, with CV 0.26%). The overall air quality was 34 AQI (32 AQI to 38 AQI, with CV 7.7%). The data details were obtained from the European Meteorological Center—ECMWF, which uses the weather forecast model HRES [34]. The duration of exposure of the material to sun or shade is given in Table 2.

A dilution of powdered acid sweat (AS) with a pH of 5.5 prepared according to BS EN ISO 105-E04 [35] was added to the specimens. The total duration of exposure to AS is shown in Table 2. After each training simulation, the polymer material was washed in a washing machine at a standard setting and a temperature of 30°. An ECE detergent (without optical bleach or phosphate) was used, according to the procedure described in EN ISO 6330: 2012 [36]. After each wash cycle (12 or 24 cycles), the material was air-dried.

### 2.2. Test Methods

In this investigation, the focus is placed on the following: polymer material topography, thickness, SEM analysis, moisture management, elongation at break, bursting force, and FTIR analysis.

#### 2.2.1. Topographic Characterization

**Figure 1:**
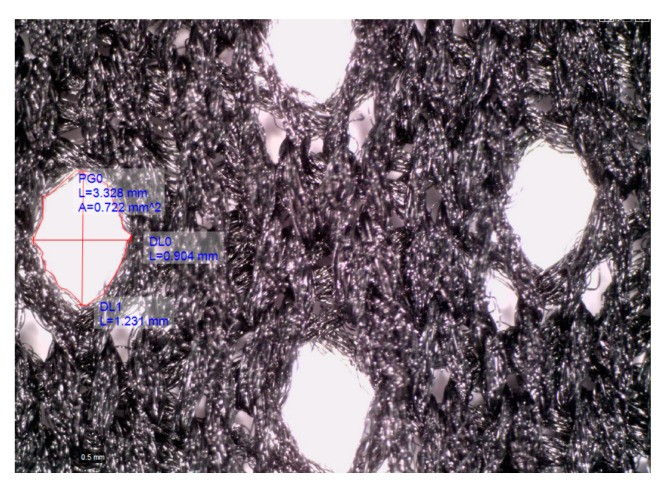
Topographic characterization in progress.

To study the topography of polymer materials, different length scales (macro and meso) have been used to interpret the changes in polymer materials due to aging. Such an approach allows characterizing the surfaces considering the specific morphologies that are established due to the type of structure, such as the dimensions of the intra yarn area (Aiy) and the appearance of the yarn structure. For each case, a different magnification and cut-off length (Lc) is defined, i.e., the length of a single side of the square sampling area. In the case of macro topography, the magnification was set to 67× and Lc to 4.0 mm; in the case of meso topography, the magnification was 167× and Lc was 1.5 mm. Topographic characterization of the polymer materials before and after aging was performed using a Dino-Lite Edge AM7915MZT digital microscope (Dino-Lite, Almere, the Netherlands). Samples of size 100 × 100 mm were prepared for analysis. Microscopic images were acquired at 10 locations along the material positioned on a flat surface in a controlled environment (20 ± 2 °C and 65 ± 2% relative humidity). Professional software DinoCapture 2.0 (Figure 1) was used for the characterization.

The fabric density, i.e., the density of stitches (main structural units that form material whose dimensions are observed lengthwise and widthwise, i.e., in the direction of courses and wales) in the horizontal and vertical directions are defined using the microscopic image analysis. In the first phase, dimensions related to the size of stitches were measured (*A*, *B*), and further calculation was conducted using the following formulas:

(1) $$D_{h}=\frac{U_{m}}{A}$$

(2) $$D_{v}=\frac{U_{m}}{B}$$

where *D_h_* is horizontal fabric density, *U_m_* is a unit of measurement, *A* is a wale spacing, *D_v_* is vertical fabric density, and *B* is a course spacing.

#### 2.2.2. Material Thickness

The thickness of the material was measured as the perpendicular distance between the reference plate on which a specimen is laid down and a parallel circular plate that covers the tested specimen under pressure, as described in ISO 5084 [37]. A thickness gauge DM-2000 (Wolf Messtechnik GmbH, Freiberg, Germany) was used for the test. The specimen was conditioned in the standard testing atmosphere before testing, as described in ISO 139 [38]. During the test, a pressure of 1 kPa was used over a specimen area of 20 cm^2^. A total of 10 measurements were taken at different locations on the specimen. The test areas were selected according to section A.3. in ISO 139 [39], i.e., the test areas were located in the central part of the specimen, arranged diagonally (starting from the left corner of the specimen). The average thickness result was expressed as the mean value of 10 measurements.

#### 2.2.3. SEM Analysis

The scanning electron microscope (FE-SEM, Mira LMU, Tescan, Brno, Czech Republic) was used for the morphological identification of the fiber surfaces. For the purpose of the scanning, the samples were coated with chrome to facilitate better specimen conductivity. The accelerating voltage was 5 kV.

#### 2.2.4. Elongation at Break

**Figure 2:**
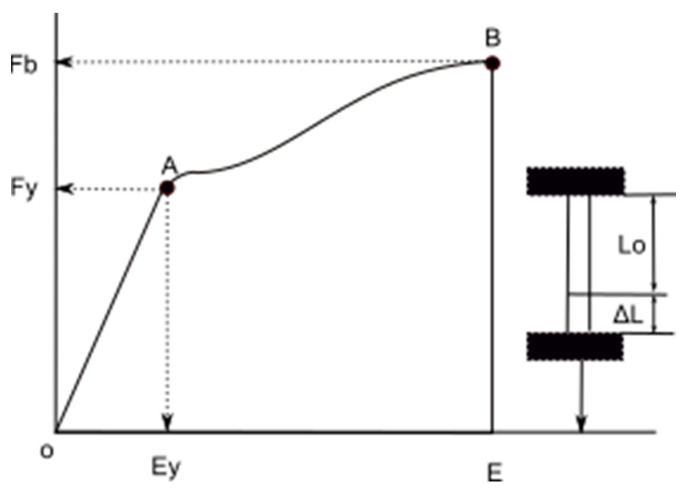
Typical load/elongation curve.

Material elongation at break (E) is defined as the elongation of a specimen corresponding to the force at the material break. When an external force is applied to a material, it is balanced by the internal force development in the material structure. A further increase in stress leads to deformation of the material, which is described by typical points (Figure 2). The region between points O and A (yield point) is the one where Hooke’s law is obeyed and is called elastic deformation. This is followed by the region between points A and B (plastic deformation), where the material deforms without the applied load increases. Point B is the breaking point where the material breaks irretrievably.

Elongation at break was tested on a Statimat M tensile tester (Textechno, Mönchengladbach, Germany) at a constant elongation rate. The strip test method was chosen, which refers to rectangular 50 × 200 mm. In accordance with Clause the B and Annex B of ISO 13934-1 [39], no specimen was cut within 150 mm of either edge of the material. The atmosphere for testing was as specified in ISO 139 [38]. On the tensile tester, the gauge length was set to 100 ± 1 mm and the test speed to 100 mm·min^−1^.

#### 2.2.5. Bursting Force

Bursting force was measured in accordance with the standard [40] using a burst tester (Apparecchi Branca, Milan, Italy) equipped with a polished steel ball that is 25.40 ± 0.005 mm in diameter and spherical within 0.005 mm. This method is ideal for measuring materials that exhibit a high degree of elongation at break, such as those selected for the investigation in this paper, and simulates the behavior of material due to intense pulling during duals of athletes in sports. During the measurement, a specimen is securely clamped without stress between grooved, circular plates of the ball burst attachment, which is attached to the pulling jaw (movable) of the testing machine at a constant traverse speed. A force is applied to the specimen by a polished, hardened ball attached to the machine’s pendulum-actuated (fixed) clamp until breakage occurs. A set of 5 circular specimens with a diameter of 50 ± 1 mm was prepared for the measurement of each polymer material. This type of measurement is independent of the direction in which the specimen is cut, since cracking naturally occurs in the direction of least resistance.

#### 2.2.6. Moisture Management

**Figure 3:**
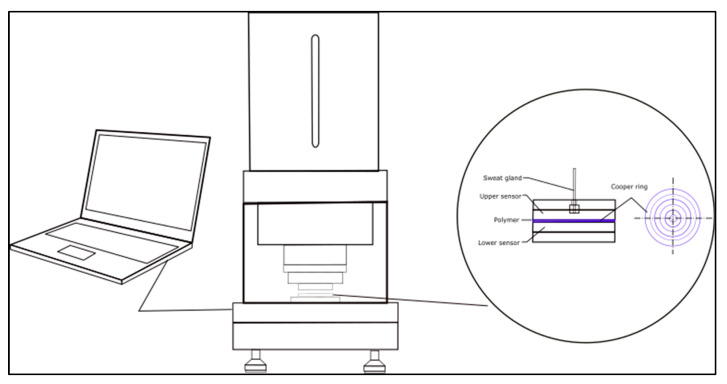
Moisture management measurement.

The parameters of moisture management were tested on the M290 moisture management tester (SDL Atlas, Rock Hill, South Carolina, USA) (see Figure 3) following the procedure described in a standard [41,42].

Among measured properties, the focus was placed on the absorption rate, spreading speed, and overall moisture management capability. The absorption rate (AR) is the average moisture absorption capacity of the material surface during the wetting process. Spreading speed (*SS*) is the cumulative spreading rate from the point where the liquid moisture is applied to the maximum wetting radius. Suppose the device (*i* = 1, 2, 3, 4, 5, 6) is wetted at time *t_i_*, the moisture spreading speed (*Si*) of liquid moisture from ring *i* − 1 to ring *i* is:

(3) $$S_{i}=\frac{\Delta R_{i}}{\Delta t_{i}}=\frac{R}{t_{i}-t_{i-1}}$$

(4) $$SS=\overset{N}{\underset{i=1}{\sum }}S_{i}=\overset{N}{\underset{i=1}{\sum }}\frac{R}{t_{i}-t_{i-1}}$$

where constant *R* is the ring radius.

Overall moisture management capability (*OMMC*) is an index that shows the capability of investigated structure to manage the transport of moisture. The index is determined using three different measured parameters, among which are moisture absorption, drying speed (both at the bottom), and one-way liquid transport capability, i.e.,

(5) $$OMMC=C_{1}\cdot AR_{B\_ndv}+C_{2}\cdot R_{ndv}+C_{3}\cdot SS_{B\_ndv}$$

(6) $$AR_{B\_ndv}=\left\{\begin{array}{cc} 1 & ,\;AR_{B}\geq AR_{B\_max}\\ \frac{AR_{B}-AR_{B\_min}}{AR_{B\_max}-AR_{B\_min}} & ,\;AR_{B}\in \;\left[ARB_{B\_min},\;AR_{B\_max}\right]\\ 0 & ,\;AR_{B}\leq AR_{B\_min} \end{array}\right.$$

(7) $$R_{ndv}=\left\{\begin{array}{cc} 1 & ,\;R\geq R_{max}\\ \frac{R-R_{min}}{R_{max}-R_{min}} & ,R\in \left[R_{min},R_{max}\right]\\ 0 & ,\;R\leq R_{min} \end{array}\right.$$

(8) $$SS_{B\_ndv}=\left\{\begin{array}{cc} 1 & ,\;SS_{B}\geq SS_{B\_max}\\ \frac{SS_{B}-SS_{B\_min}}{SS_{B\_max}-SS_{B\_min}} & ,SS_{B}\in \left[SS_{B\_min},SS_{B\_max}\right]\\ 0 & ,SS_{B}\leq SS_{B\_min} \end{array}\right.$$

where *OMMC* is overall moisture management capability; *AR_B_* is absorption rate; *R* is one-way transport capability; *C*_1_, *C*_2_, and *C*_3_ are the weighting values for *AR_B_ndv_*, *R_ndv,_* and *SS_ndv_*; *AR_B_max_*, *AR_B_min_*, *R_max_*_,_ *R_min_*, *SS_max_*, and *SS_min_* are the maximum and minimum values for each property.

#### 2.2.7. FTIR Analysis

Attenuated Total Reflectance (ATR)-FTIR spectra were recorded using Perkin Elmer Spectrometer—Spectrum Two (Perkin Elmer, Waltham, MA, USA), performing 10 scans in a range of 4000 to 450 cm^−1^ with a spectral resolution of 4 cm^−1^. The pressure was tenably adjusted to 90–95% strength to obtain spectra with constant penetration depth to the textile piece, using a 250 × 250 mm area of the sample. FT-IR spectra of starting sample, as well as spectra of the samples aging in the sun or shade, are shown in Supplementary Materials (Figures S1–S15).

## 3. Results and Discussion

### 3.1. Results of the Topographic Characterization

**Figure 4:**
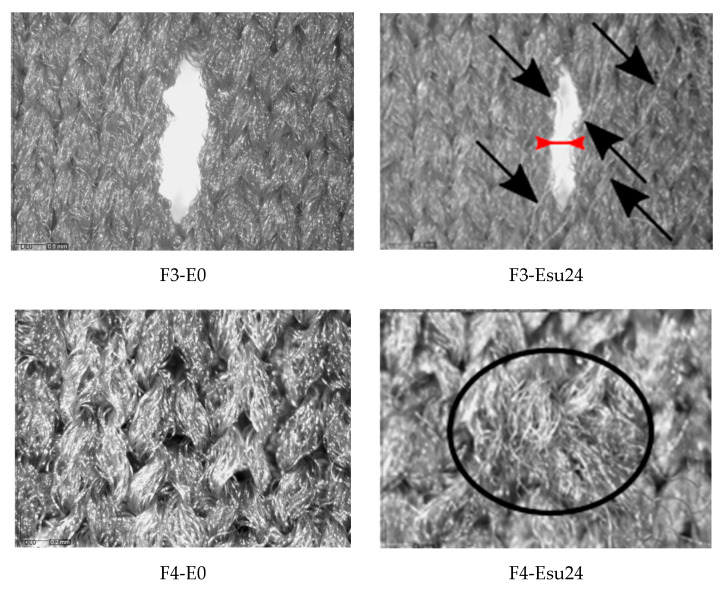
The microscopic images of polymer materials F3 and F4—non-exposed (E0) and after the longest exposure to aging, including the exposure to the sun (Esu24).

The examples of microscopic images of non-aged materials and materials aged according to Esu24 are given in Figure 4. The examples are given for specimens F3 and F4, which represent different structure types; the changes in the visual appearance are well perceived. From the images of both samples exposed to the sun, the increase in the surface fibrillation (additionally pointed out in the image using the black arrows) is seen. The primary reason for the splitting of the fibril bundles and their subsequent exposure to the surface is the conducted and repeated care process. This investigation was able to confirm the additional influence of exposure to the sun on the increase in fibrillation because the fibrillation was more prominent on the surface of materials exposed to the sun than those exposed to shade. The observation brings us to the conclusion that the conducted process of aging negatively affected the visual aspect of the material surface. This phenomenon should further lead to consumer dissatisfaction with the material appearance. The images also illustrate the change in the dimensions of voids within the structure, which is especially well seen if the specimens F3-E0 and F3-Esu24 are compared. The decrease in the void width is marked on specimen F3-Esu24 using the red arrow. For these particular cases, the maximal width of the unexposed specimen (i.e., F3-E0) is 0.794 mm; the maximal length of the void of the specimen exposed to the sun (i.e., F3-Esu24) is 0.442 mm. Regarding the length of the observed voids, the decrease is also confirmed after the exposure to the sun (for example, the length of the void of the unexposed specimen F3 is 2.894 mm, while the length of the specimen F3 exposed to the sun is 2.569 mm). These observations lead to the conclusion that the aging of materials has caused the material shrinkage in both dimensions (i.e., lengthwise and widthwise).

The numerical results of Aiy (Table 3) have confirmed the instability of the structure and transformation of Aiy affected by aging. The average values of Aiy before aging are 0.010 mm^2^ to 1.762 mm^2^ (i.e., 0.01–0.029 mm^2^ for specimens without holes and 0.722–1.762 mm^2^ for specimens with holes). The exposure to Esu24 caused a decrease in the original Aiy by up to 20%, with the highest decrease for specimen F4. The values of Aiy after aging Esh24 are 0.009 mm^2^ to 1.458 mm^2^, again with the highest decrease for specimen F4. In contrast to earlier findings where the influence of individual aging in the changes in topology was not observed, the results of this study indicated the decrease in Aiy due to individual aging for all measured samples, as well as a slight, but consistent decrease in Aiy for Esu24 than Esh24.

The previously described changes can be explained by the dimensional change of a basic structural unit, which requires the need to further investigate the horizontal and vertical density of structural units; therefore, the changes in horizontal and vertical density compared to the densities of non-aged specimens, are presented in Table 4. As can be seen from the results, for all the observed specimens, there is a positive trend of increase in horizontal and vertical density after exposure for all the samples studied. This is consistent with previous conclusions regarding visually observed changes in void dimensions as well as Aiy. All of the previously described changes should be explained by the fact that the aging performed resulted in a shrinkage of the material. Knitted fabrics are known to be easily deformed due to the specific shape of the structural units. As reported in the literature, the dimensions of such fabrics expand during the process of material production [43] and shrink during finishing processes [44]. The results of the analysis described in this section have shown that all specimens, regardless of the type of structure (double jersey, double jersey with holes, or double jersey plated), exhibit structural changes, i.e., shrinkage due to aging.

### 3.2. Results of Thickness Testing

**Figure 5:**
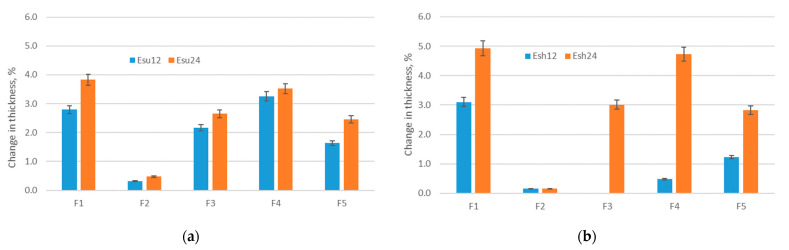
Change in material thickness due to exposure: (**a**) Esu; (**b**) Esh.

The results of thickness testing (Figure 5) revealed the increase in thickness due to exposure to the sun for all investigated materials. As can be seen, the thickness of materials increases after the exposure to Esu12 (increase is 0.4–3.1%) and continues to increase after exposure to Esu24, (the increase is 0.5–3.9% in comparison to the primary thickness). The main reason for the increase in material thickness is the material shrinkage confirmed in the previous section, which for a range of elastic materials with an unstable structure, directly affects the change of the observed property.

Regarding the influence of different types of structures (D, DH, DP), the highest differences within the observed values are for the structures with integrated holes that are evenly distributed within the structure (structure type DH, material F1). In this case, the reason is the change of the void dimensions that enabled the more prominent increase in structure density. As a result, the surface of the area of voids without yarns decreased and was partially replaced with yarns forming the stitches; therefore, instead of a yarn-free area, the thickness tester was able to measure the thickness of yarn instead. As opposed to the behavior of the DH type of the structure, the double jersey structure remained with minimal changes in thickness, which brings us to the conclusion that this structure is most stable when exposed to different aging factors, including exposure to the sun. Unlike exposure to the sun, the exposure of polymer materials to aging in the shade did not indicate an unambiguous change of thickness after exposure Esh12; however, the thickness increases after the exposure to Esh24 and follows the values of thickness after Esu24. Regarding the impact of the structure, the structure F1 again exhibited the highest differences within the observed values. For one specimen only (F3-Esh12), there was no change in thickness due to aging.

### 3.3. Results of SEM Analysis

**Figure 6:**
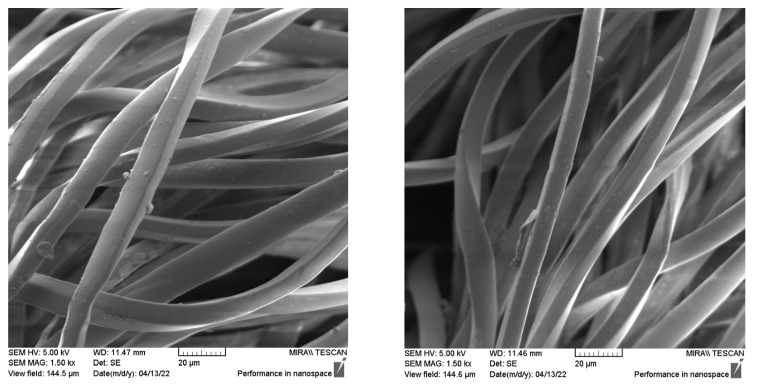
SEM images of F4-Esu24 under magnification of 1.50 kx.

SEM images of investigated sample F4 after aging Esu24 are presented in Figure 6. Presented images are representative of the whole set of investigated materials. SEM analysis indicated an existing deposition of substances on the fiber surface. Those substances are most likely to be parts of detergents and limescale due to the fact that the hard water was used (precisely, the water used for the care process had an average of 15° dH). The images were compared to the images presented within the investigation that was focused on the deteriorated polymers [45]. In the mentioned study, the images showed numerous cracks often bridged by ‘nanofilaments’, which is not the case with the images presented in Figure 6. This leads to the conclusion that there was no polyester degradation in his phase that solar radiation could bring.

### 3.4. Results of Elongation at Break Testing

**Figure 7:**
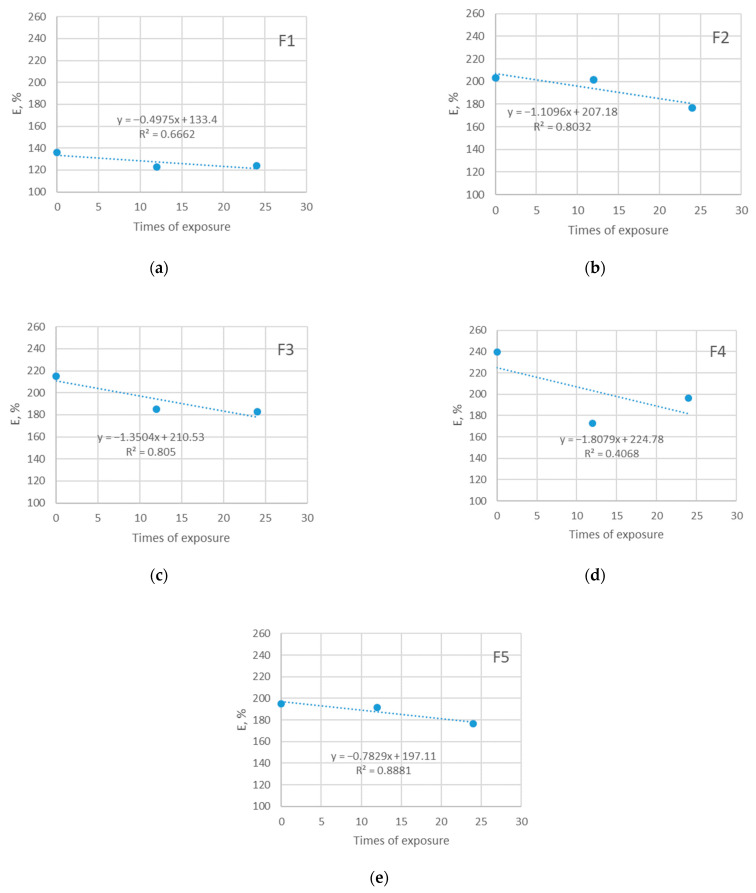
Breaking elongation of five investigated materials exposed to sun: (**a**) F1; (**b**) F2; (**c**) F3; (**d**) F4; (**e**) F5.

**Figure 8:**
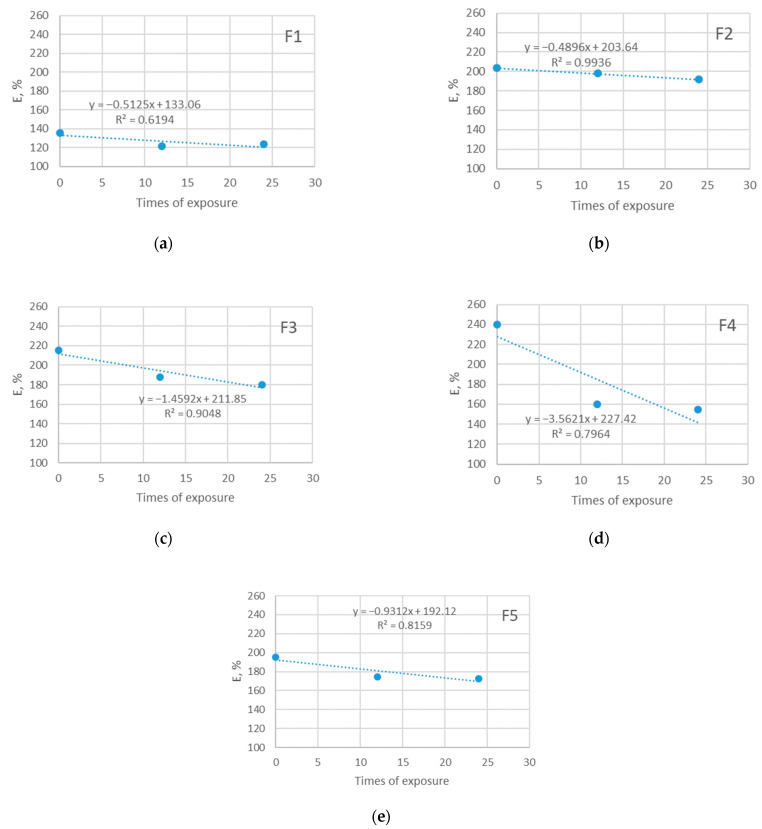
Breaking elongation of five investigated materials exposed in shade: (**a**) F1; (**b**) F2; (**c**) F3; (**d**) F4; (**e**) F5.

The instability of the knitted structure and ability to elongate allows it to fit the human body better and to give an adequate response to the pulling that happens during sports matches. The current study confirms previous research in this area. As can be seen from Figure 7 and Figure 8, the ability of materials to elongate until the moment of rupture decreases due to exposure to the sun. The elongation of investigated materials before exposure is in the range 138–240% and it decreases to the values of 120–138% after the longest period of exposure.

Broadly speaking, the decrease in elongation among the materials that were not exposed to the sun follows a similar pattern, as does the decrease in elongation of materials exposed to the sun. The observation implicates that exposure to the sun does not have an additional stronger impact on the elongation of materials. This can be explained by the fact that there was no polyester degradation in his phase (confirmed by the SEM analysis) that solar radiation could bring; however, it is expected that further prolonged exposure to sunlight may cause such degradation, which will be investigated in future research. The reason for the observed decrease in material elongation is likely the combined effect of all aging parameters (i.e., both care and outdoor exposure). Since the analysis of SEM indicated an existing deposition of detergents and limescale on the fiber surface, it is assumed that this is the factor that affected the behavior of the material, leading to a decrease in elasticity.

For the majority of cases, the relationship between the observed variable and times of exposure is strong (R^2^ reaches up to 0.99). The exception is the material F1 with a moderate relationship to the observed (R^2^ is 0.66 and 0.62).

**Figure 9:**
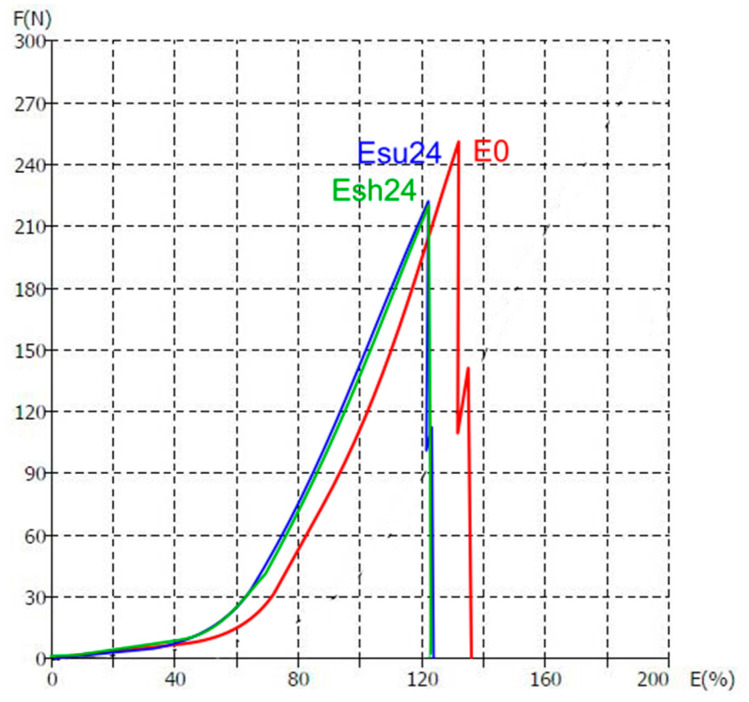
The F/E curves for the specimen F1 in the conditions E0, Esu24, and Esh24.

Figure 9 gives the typical F/E curves for the specimen F1 in conditions E0, Esu24, and Esh24. It can be seen immediately that the yield elongation for non-exposed material is slightly higher than for exposed to aging material. It corresponds to an estimated elongation of 50% for non-exposed and 40% for exposed materials. Breaking elongation for non-exposed material is 135.84% and is 9% higher than for exposed materials. When we look at forces, there are no significant differences for yield forces; however, there is a difference in breaking force. For non-exposed material, it is 254.64 N and is on average 12% higher than the force for exposed materials. Interestingly, the values for exposed materials are uniform and there is no significant difference between material exposed to the sun from material aged in the shade.

### 3.5. Results of Bursting Force Testing

The stresses that may be applied to the polymers used for the purpose, such as the one defined for the specimens investigated within this paper, could be, on certain occasions, significant; therefore the insight into the changes of this property is important for the description of the long-lasting performance of polymer material. A significant decrease in PES force due to aging has been reported by scientists [1,2,17,18], but those results did not focus on the changes of forces due to the specific type of stress, such as bursting.

**Figure 10:**
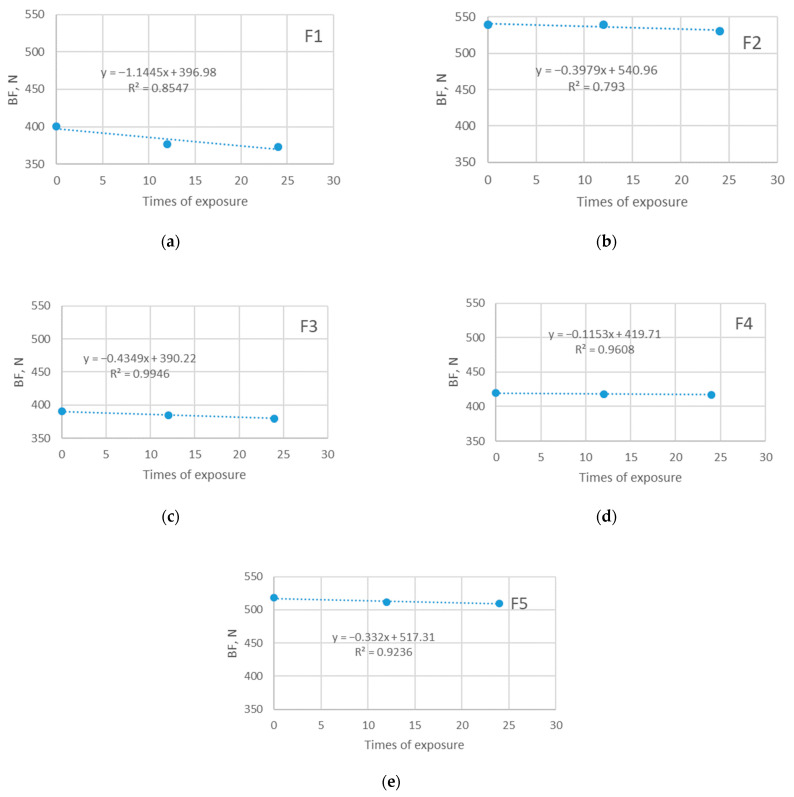
Bursting force (BF) of five investigated materials exposed in sun: (**a**) F1; (**b**) F2; (**c**) F3; (**d**) F4; (**e**) F5.

**Figure 11:**
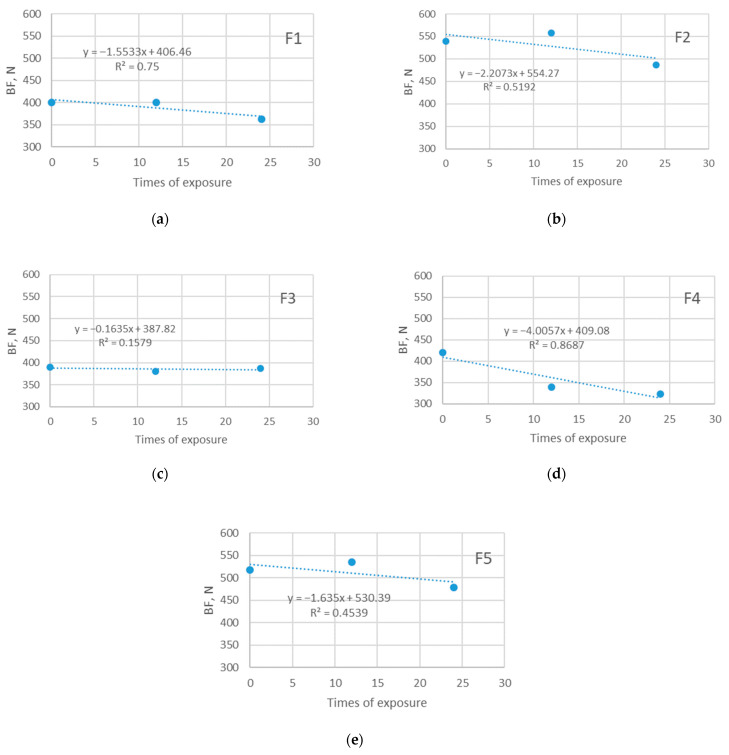
Bursting force of five investigated materials exposed in shade: (**a**) F1; (**b**) F2; (**c**) F3; (**d**) F4; (**e**) F5.

The results of bursting force testing are given in Figure 10 and Figure 11. The single graphs presented in Figure 10 offer compelling evidence for the impact of exposure to the sun on the changes in the values of the bursting force. As can be seen, the initial values are in the range 390–539 N. Over time, the exposure to the sun influences the decrease in bursting force, so the final values after Esu24 are in the range 372–530 N, which presents a decrease of up to 6.8%. Although notable, this decrease does not indicate that the durability of polymer material is strongly disturbed, as was shown in previously published studies, where the decrease due to aging was up to 40% [17]. The decrease in bursting force is evident in all cases (i.e., for all specimens tested). As can be seen from the graphical presentation, there is a strong negative correlation between the exposure time and bursting force with corresponding linear regression coefficients higher than 0.793.

Interestingly, a substantial disagreement between the periods of exposure and measured breaking forces is seen for the specimens exposed to Esh12 and Esh24. The results presented in Figure 11 show a slight increase in bursting force after exposure to Esu12 for three out of five specimens, which cannot be related to the structure, thickness, or Aiy. Still, the finally measured bursting force (i.e., the bursting force measured after Esh24) decreases in all cases and is lower than the bursting force of the unexposed specimens. The apparent lack of correlation between the time of exposure and bursting force is confirmed by linear regression equations (the relation is strong for one specimen only—R^2^ for the F4 is 0.8687).

### 3.6. Results of Moisture Management Testing

**Figure 12:**
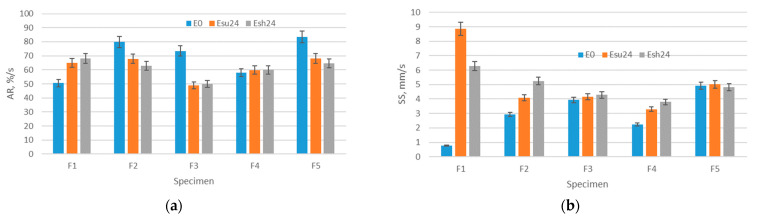
Average values and standard deviation of the properties related to moisture management: (**a**) AR and (**b**) SS of polymer materials.

The results of moisture management testing, including AR, SS, and OMMC are shown in Figure 12. As seen in Figure 12a, the values of AR are in the range of 50 %/s to 83 %/s. Interestingly, the results of the absorption rate testing revealed different trends of values due to aging for the observed specimens. Precisely, the absorption rate for specimens F2, F3, and F5 decreased due to aging. This decrease indicates the significantly impaired capacity of the polymer material to absorb moisture. In contrast, the absorption rate of specimen F1 increased due to aging from the initial value of 50 %/s to 68%/s for the aging Esh24. The reason for this rather contradictory result should be explained by the detected significant change in material thickness, which enabled a higher absorption of moisture on the surface of this specimen. Nevertheless, the statistical analysis conducted in the Statistica software, taking all specimens into account, confirmed that the correlation between the thickness and absorption rate is rather strong (correlation coefficient is 0.897; significant at *p* < 0.0500). In contrast, the trend of SS decrease is well seen in Figure 10b. The values of SS in the state E0 are in the range 0.7–4.9 mm/s and increase after aging from 3.3 mm/s to a maximum of 8.8 mm/s (observed for F1 after Esu24). Again, the specimen F1 stands out due to the highest increase in SS, which should be explained by a noted increase in thickness, but also high differences within the Aiy values that described the behavior of the structure with integrated holes that are evenly distributed.

**Figure 13:**
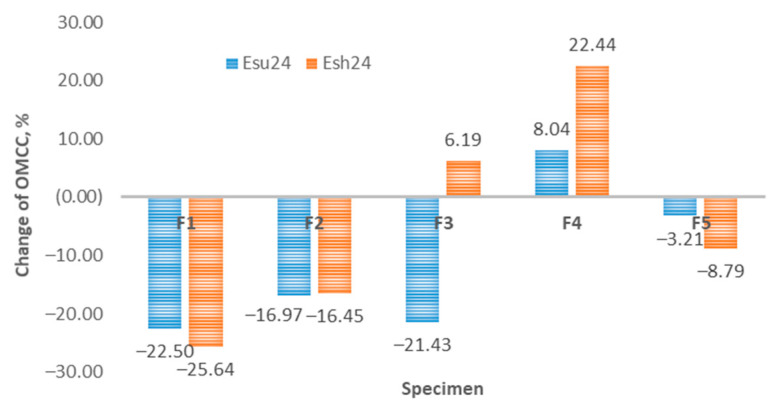
Change of OMMC due to aging.

Figure 13 shows the comparative aging results after Esu24 and Esh24 and their impact on the total moisture management capacity (OMMC) for specimens F1-F5. The greatest effect of aging on the OMMC index is seen on specimens F1 and F2, in the percentage ranges of 16% to 26%, and with minimal difference in relation to aging Esu24 and Esh24. This means that the impact of solar radiation on the OMMC index on these two samples is minimal compared to shadow aging. The aging results on sample F3 are contradictory. Namely, sun exposure (Esu24) shows a decrease in the OMMC index of approximately 20% (same range as for samples F1 and F2), while shadow aging (Esh24) shows an increase in OMMC index by 6%. Sample F4 shows an increase in the value of the OMMC index after exposure to aging—an 8% increase after Esu24 aging and a 22% increase after Esh24 aging. The change in the percentage of OMMC for sample F5, after the aging process, shows a decrease of 3–9%, with a greater decrease after Esh24 aging. The measurement results for samples F1, F2, F3, and F5 show a decrease in the percentage of OMMC index after Esu24 aging. Interestingly, the increase was detected only for sample F4 (after Esu24 and Esh24) and for sample F3 only after Esh24. This increase in OMMC for specimens F4 and F3 (Esh24 only), may be associated with a significant increase in thickness (compared Esh12 and Esh24) for specimens F3 and F4. Figure 5 shows a significant increase in thickness as a result of Esh24. Higher thickness increased the absorption rate of specimen AR, and thus OMMC.

### 3.7. Results of FTIR Analysis

Owing to its chemical origin, all samples showed a characteristic IR-absorption band of the carbonyl group from the ester link at 1712 cm^−1^. Since the structure of PET polyester is well known, we found that all other absorption bands are completely correlated with the standard IR spectra of polyester [27,46,47]. Predominant absorption bands are detected at 1408, 1339, 1239, 1093, 1016, 871, and 722 cm^−1^ and run out from stretching and bending vibrations of the corresponding methylene, terephthalate, and ester bonds (see Supplementary Materials).

According to the findings in the literature, changes observed in IR spectra during the experiment are usually caused by chemical structure modifications; for example, ATR-FTIR was a method of choice to identify the oil as a polyurethane breakdown product [8]. Marjo et al. tested the chemical hydrolysis of seven garments, demonstrated by ATR-IR, which showed that two items had a vulnerable polyester copolymer, and one item had a chemically resistant polyether copolymer. Cai et al. employed FTIR to characterize the reaction mechanism between citric acid with cellulose in cotton fabrics to determine the changing sequence order of citric acid from anhydride and unsaturated acid (the reason for yellowing) [48]. Du et al. revealed a method based on the acid/ester ratio from the carbonyl region in IR spectra, which proved to be a good indicative parameter of the chain scission during hydrolysis degradation of other polyesters [24]. Partini et al. analyzed the hydrolysis of PET in an alkaline atmosphere, presenting that the band at ~1570 cm^−1^ shows the degradation point originated from carboxylate that is formed during hydrolysis [49]. In our previous study, we also used the FTIR method to evaluate the aging of PA and PES materials, demonstrating the physicochemical changes in the surface of the PA material by the appearance of a new peak in the carbonyl region [17].

**Figure 14:**
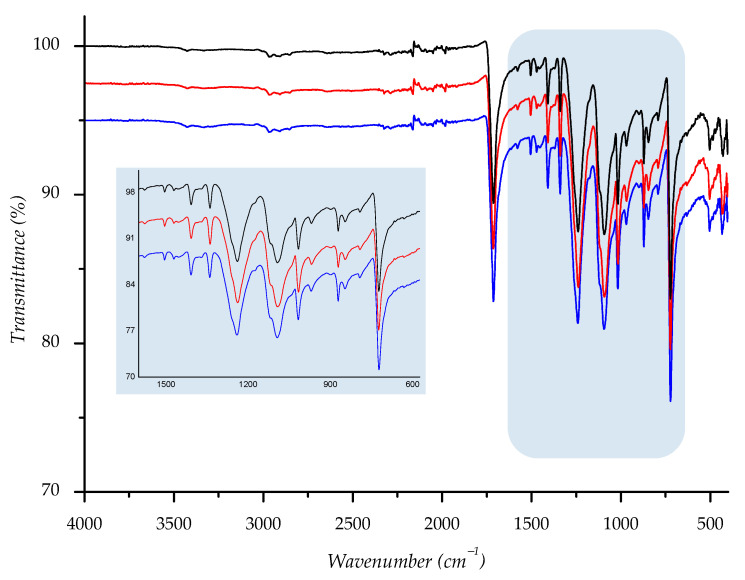
IR spectra of investigated sample F1 ((—) = starting material; (—) = material aged in sun; (—) = material aged in shade) with enlarged fingerprint region (colored in blue).

**Figure 15:**
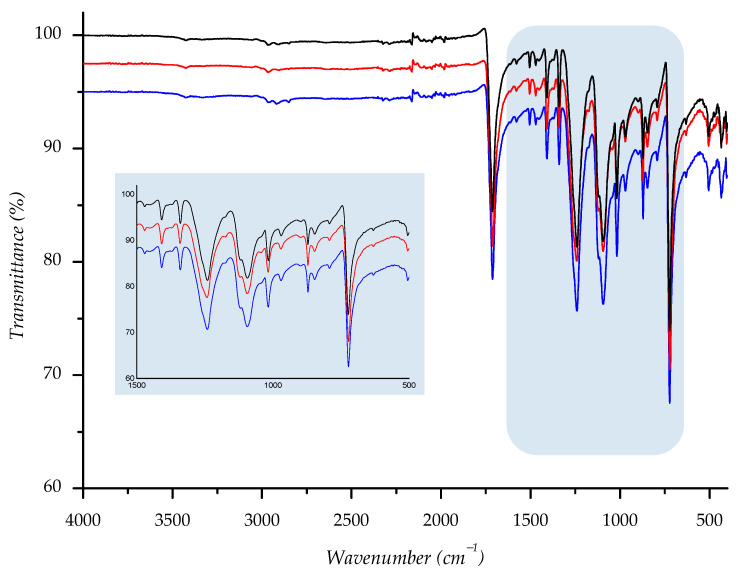
IR spectra of investigated sample F2 ((—) = starting material; (—) = material aged in sun; (—) = material aged in shade) with enlarged fingerprint region (colored in blue).

**Figure 16:**
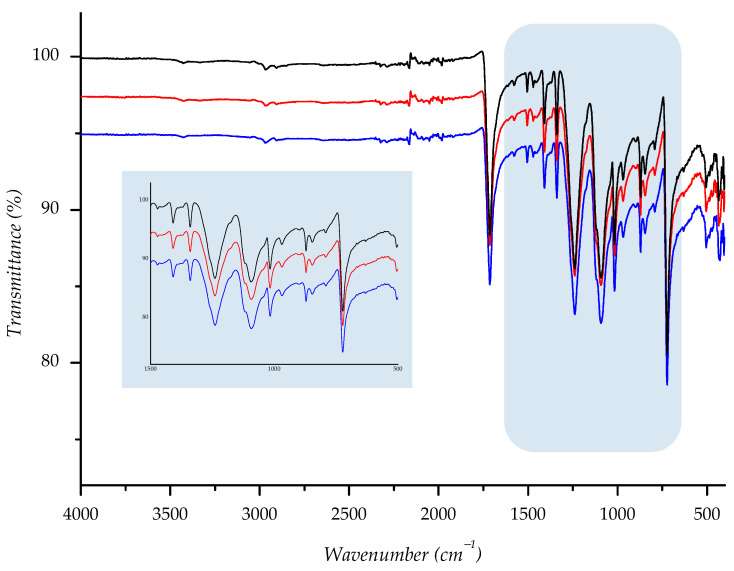
IR spectra of investigated sample F3 ((—) = starting material; (—) = material aged in sun; (—) = material aged in shade) with enlarged fingerprint region (colored in blue).

**Figure 17:**
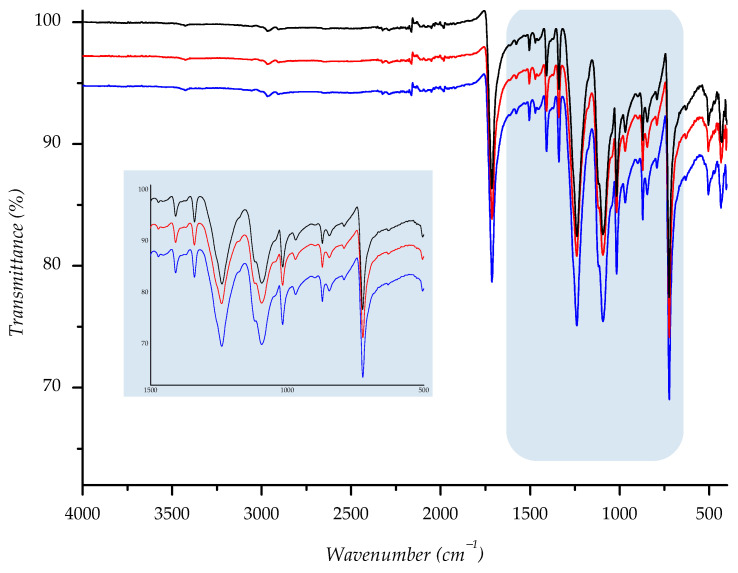
IR spectra of investigated sample F4 ((—) = starting material; (—) = material aged in sun; (—) = material aged in shade) with enlarged fingerprint region (colored in blue).

**Figure 18:**
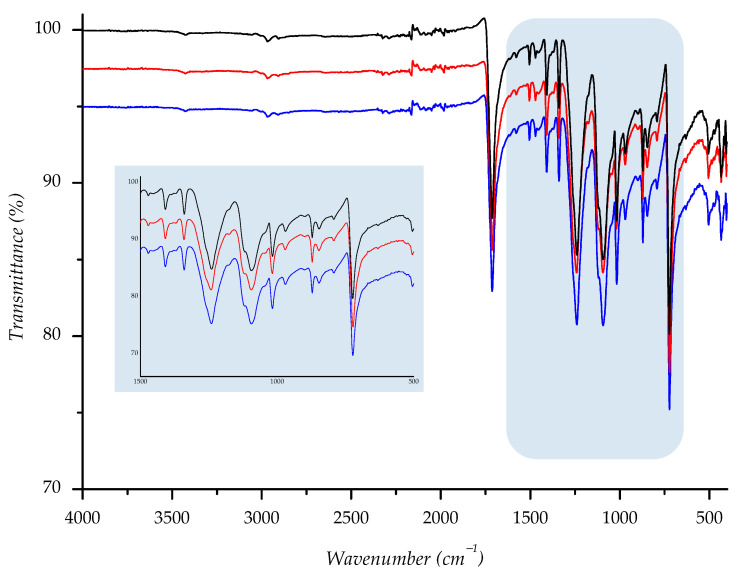
IR spectra of investigated sample F5 ((—) = starting material; (—) = material aged in sun; (—) = material aged in shade) with enlarged fingerprint region (colored in blue).

To compare spectral data of normal aging samples to ones aged in sun/shade, we overlaid spectra; the derived combination is shown below (Figure 14, Figure 15, Figure 16, Figure 17 and Figure 18). As can be seen from the spectra, the starting sample could not be distinguished from the two others, indicating that there is no detectable discrepancy between the starting and aged samples, since there were no chemical changes during the aging.

## 4. Conclusions

In this paper, we presented a new approach to polymer aging and applied it to examine the change of a set of properties, among which the structure topography, thickness, moisture management, elongation at break, and bursting force. The evidence from this study implies that the thickness of polymer materials increases due to exposure to the sun, which is to be associated with material shrinkage. The findings of this study indicate the decrease in both the ability to elongate and bursting force due to aging, which directly affects the performance of polymer materials. In terms of absorption ability, the result suggests the significantly impaired capacity of the polymer material to absorb moisture, which is negatively related to the perception of comfort. Still, in terms of the chemical structure, the results obtained using the ATR-FTIR (attenuated total reflectance) method showed that there was no detectable influence of aging (either in the sun or in the shade) on the chemical structure of polyester samples. SEM analysis indicated an existing deposit of substances on the fiber surface (most likely parts of detergents and limescale) and confirmed that there was no polyester degradation in this phase that solar radiation could cause.

Taken together, the results indicated unwanted changes in the observed properties, as well as highlighted a need to obtain an insight into the behavior of polymer material under specific conditions (i.e., those that are characteristic for each type of product or activity). The study has made steps towards enhancing the understanding of aging polymer materials used for sports. The aging protocol proposed is to be further used for any kind of sportswear aging, with the update of parameters that are characteristic of a specific sport. The future investigation is to be directed towards a longer period of exposure to determine the needed period for the material degradation, as well as the comparison to the aging of specimens aged in a controlled environment.

## Figures and Tables

**Table 1:** Material description.

Material ID	Type	Mass, g/m^2^		Yarn Fineness, Tex
		Avg., g/m^2^	CV, %	
F1	DH	105.91	3.13	12
F2	D	152.95	0.14	12
F3	DH	192.09	2.66	12
F4	DP	157.82	3.65	15
F5	DP	136.39	3.27	12

Legend: D—double jersey, DH—double jersey with holes; DP—double jersey plated.

**Table 2:** Overview of the aging parameters.

Designation	Weathering Exposure	Total Duration, min	Artificial AS Exposure	Total Duration, min
E0	-	0	-	0
Esu12	+sun	180	+	540
Esu24	+sun	360	+	1080
Esh12	+shade	180	+	540
Esh24	+shade	360	+	1080

**Table 3:** The average values of the intra yarn area (Aiy).

Material ID	Aiy					
	E0		Esu24		Esh24	
	Avg., mm^2^	CV, %	Avg., mm^2^	CV, %	Avg., mm^2^	CV, %
F1	0.722	2.24	0.625	3.52	0.651	3.81
F2	0.029	0.98	0.024	1.30	0.024	1.11
F3	1.762	2.12	1.429	4.23	1.458	4.11
F4	0.010	0.67	0.008	1.35	0.009	1.31
F5	0.021	0.55	0.018	1.08	0.019	0.98

**Table 4:** The changes in horizontal (*D_h_*) and vertical density (*D_v_*) due to aging (in %).

Material ID	*D_h_*		*D_v_*	
	Esu24	Esh24	Esu24	Esh24
F1	3.9	3.5	3.2	3.3
F2	0.6	0.5	0.4	0.0
F3	1.4	2.6	1.2	0.7
F4	2.4	2.4	2.2	2.2
F5	1.6	1.3	1.6	1.1

## Data Availability

Not applicable.

## Supplementary Materials

The following supporting information can be downloaded at: https://www.mdpi.com/article/10.3390/polym14091682/s1, Figure S1: IR spectrum of compound F1Ø; Figure S2: IR spectrum of compound F1su24; Figure S3: IR spectrum of compound F1sh24; Figure S4: IR spectrum of compound F2Ø; Figure S5: IR spectrum of compound F2su24; Figure S6: IR spectrum of compound F2sh24; Figure S7: IR spectrum of compound F3Ø; Figure S8: IR spectrum of compound F3su24; Figure S9: IR spectrum of compound F3sh24; Figure S10: IR spectrum of compound F4Ø; Figure S11: IR spectrum of compound F4su24; Figure S12: IR spectrum of compound F4sh24; Figure S13: IR spectrum of compound F5Ø; Figure S14: IR spectrum of compound F5su24; Figure S15: IR spectrum of compound F5sh24.
