# Supplementary material for: Evaluation of Changes in Polymer Material Properties Due to Aging in Different Environments
Source: Polymers (Basel). 2022 Apr 21;14(9):1682. doi: 10.3390/polym14091682 (PMC9102830; doi:10.3390/polym14091682)
Supplement: Supplementary file 1 [file polymers-14-01682-s001.zip › polymers-1668168-supplementary.pdf]

Supplementary Material

Content:

IR spectra **2-16**

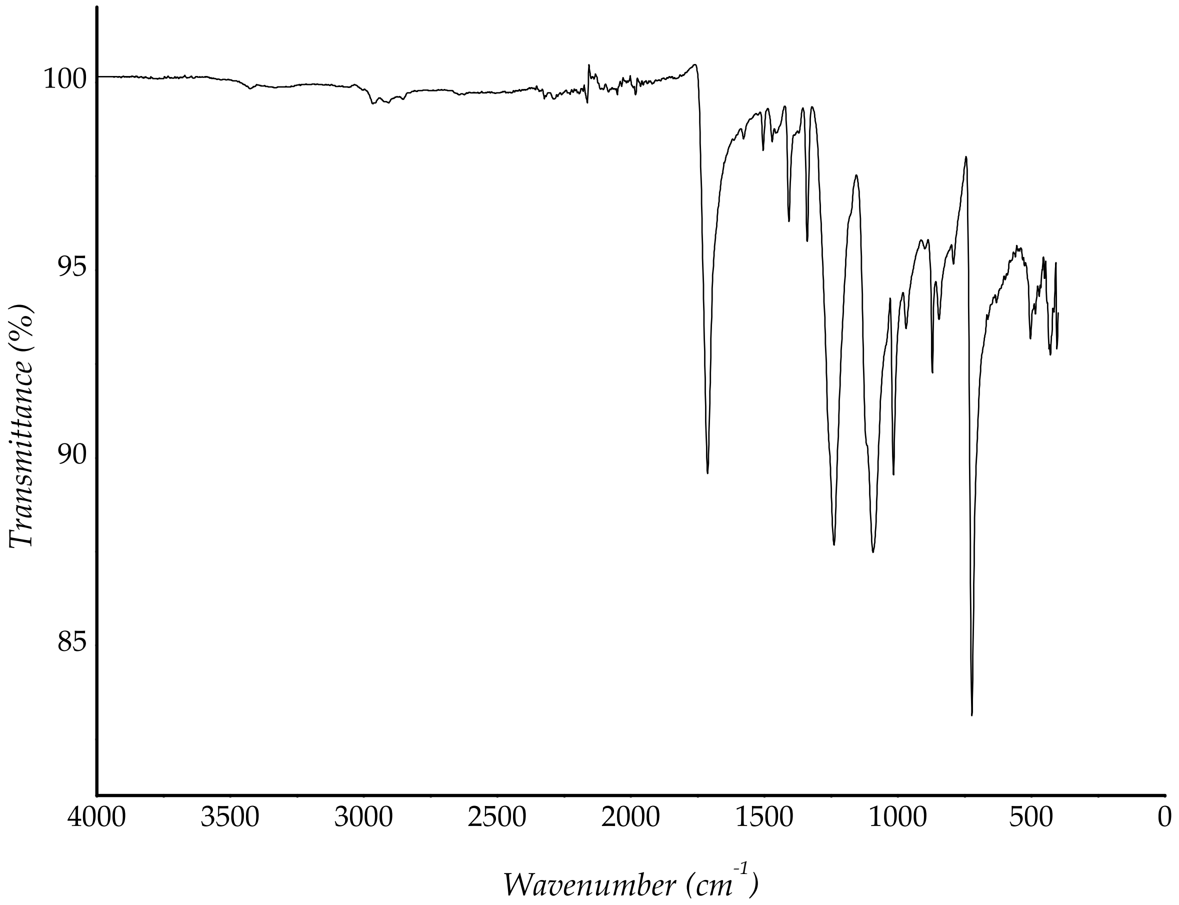

Figure S1. IR spectrum of compound **F1Ø**

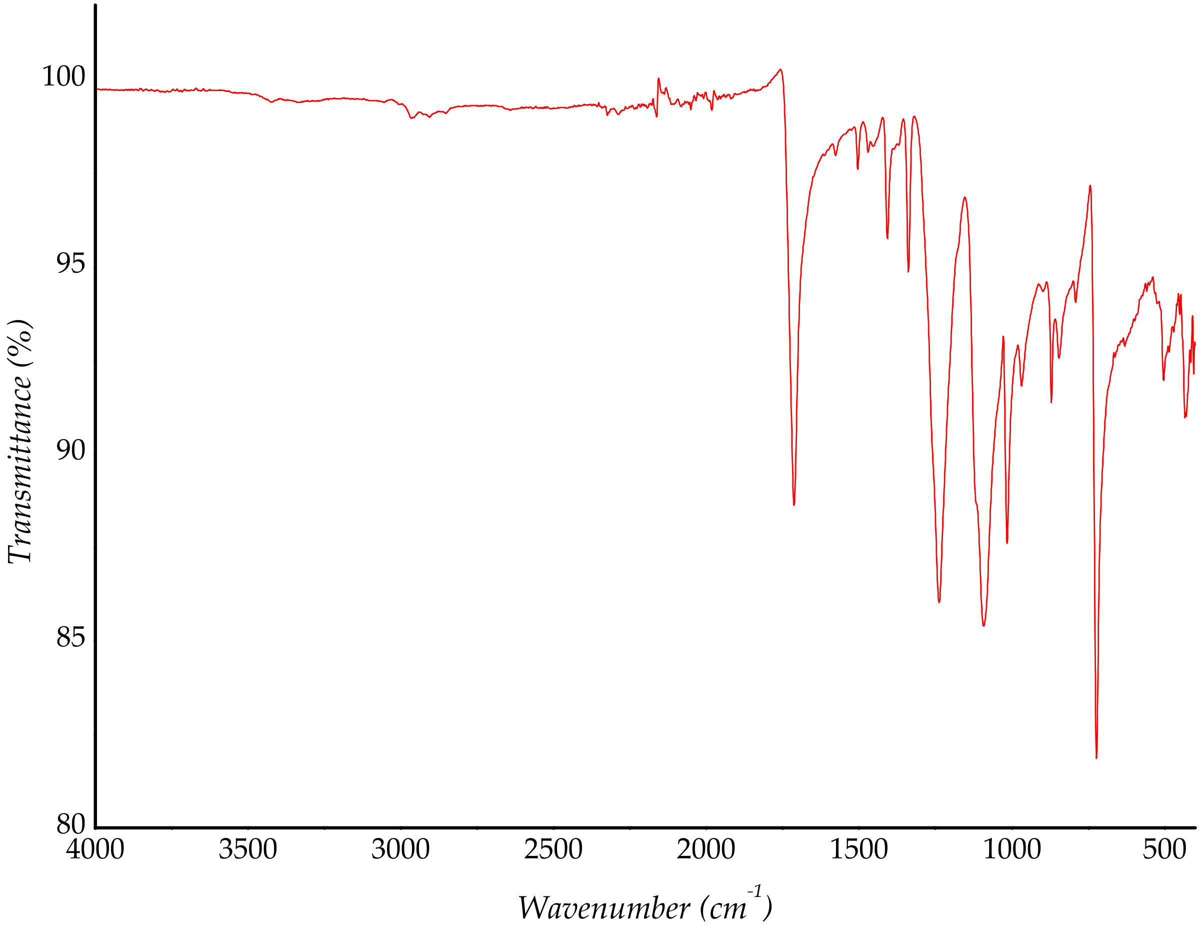

Figure S2. IR spectrum of compound **F1su24**

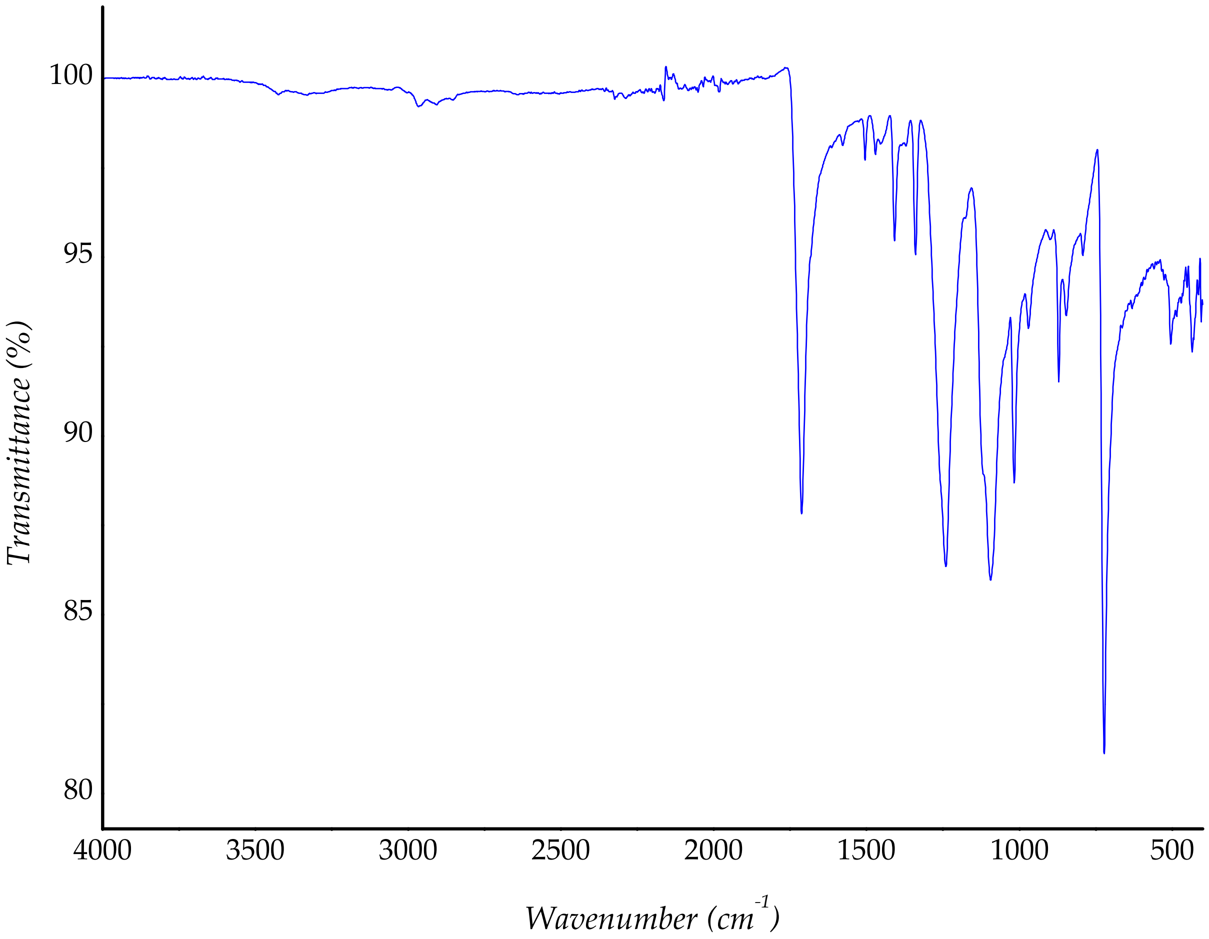

Figure S3. IR spectrum of compound **F1sh24**

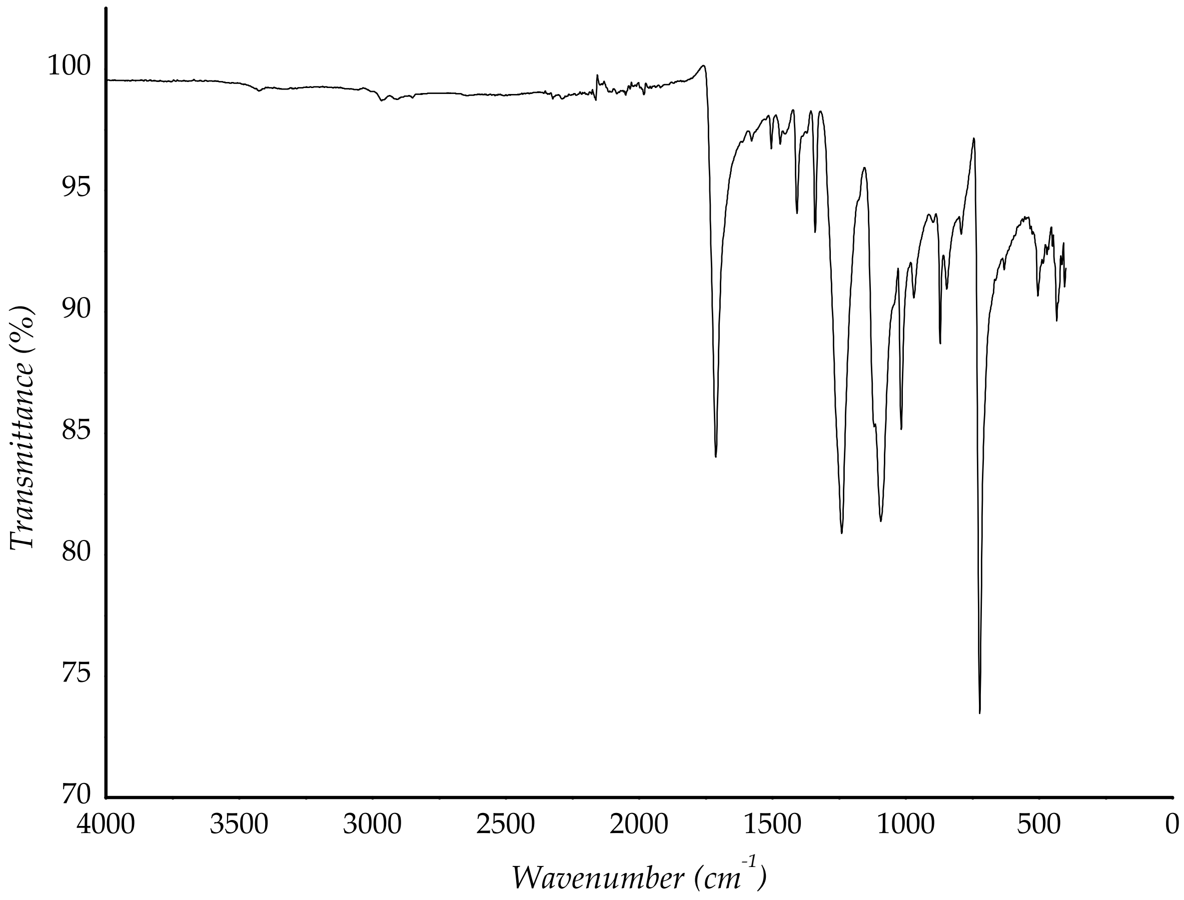

Figure S4. IR spectrum of compound **F2Ø**

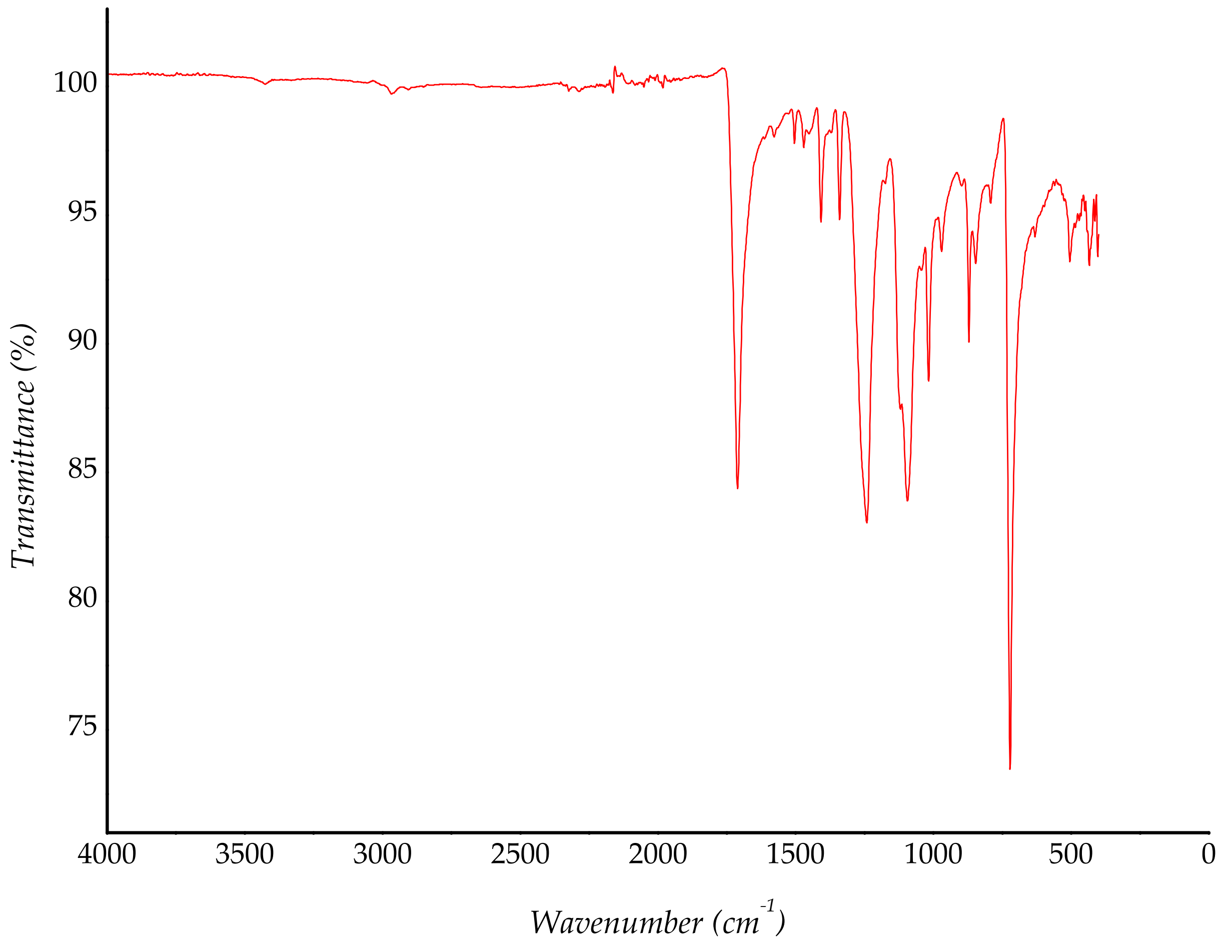

Figure S5. IR spectrum of compound **F2su24**

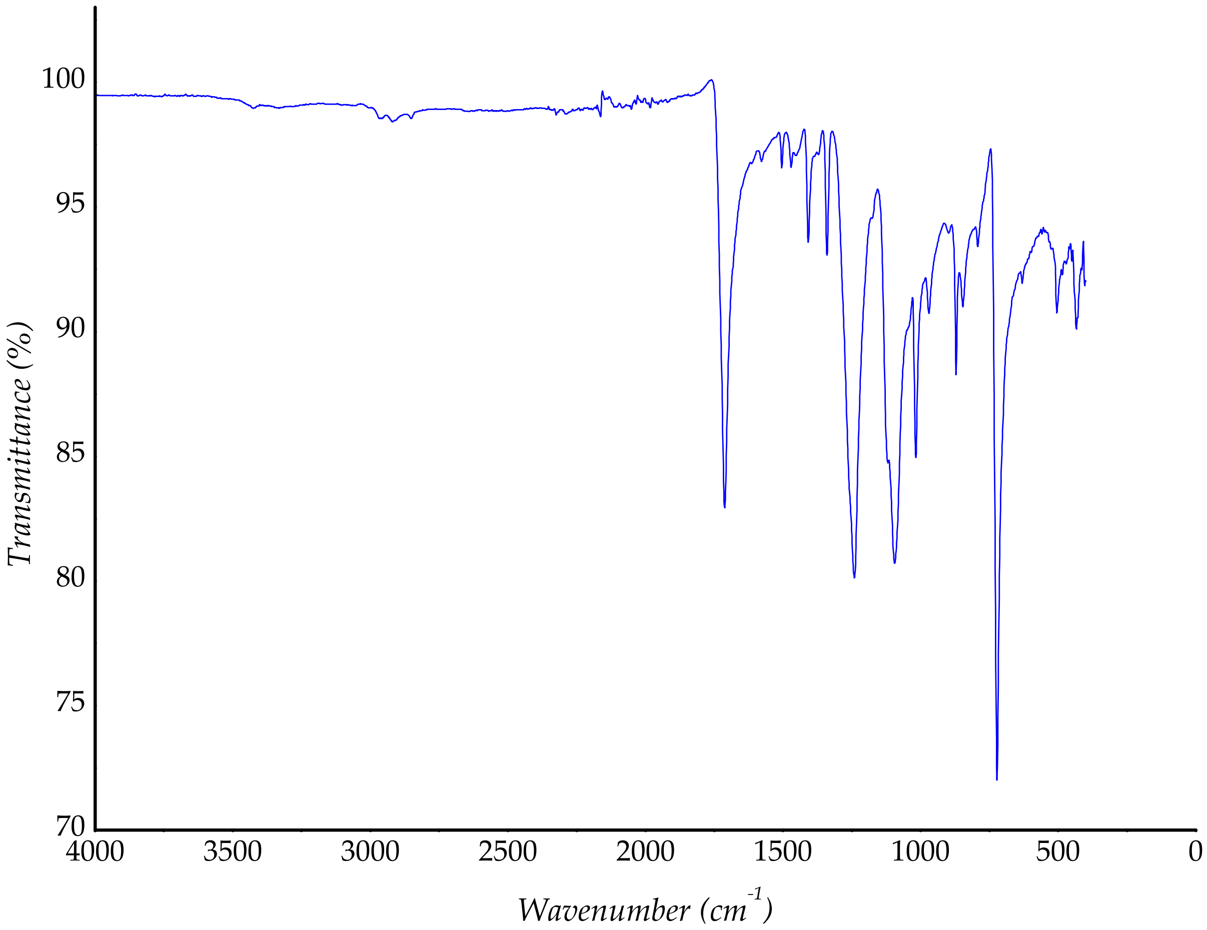

Figure S6. IR spectrum of compound **F2sh24**

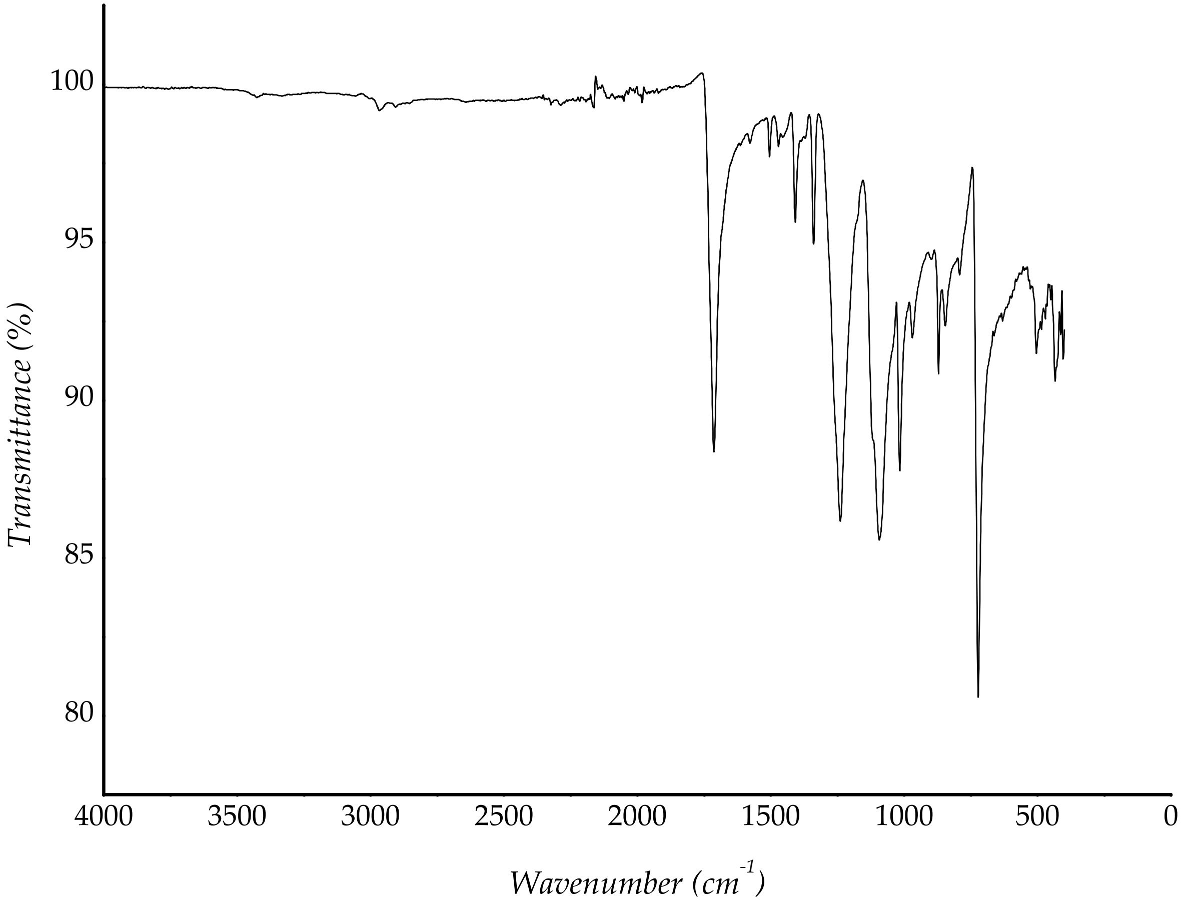

Figure S7. IR spectrum of compound **F3Ø**

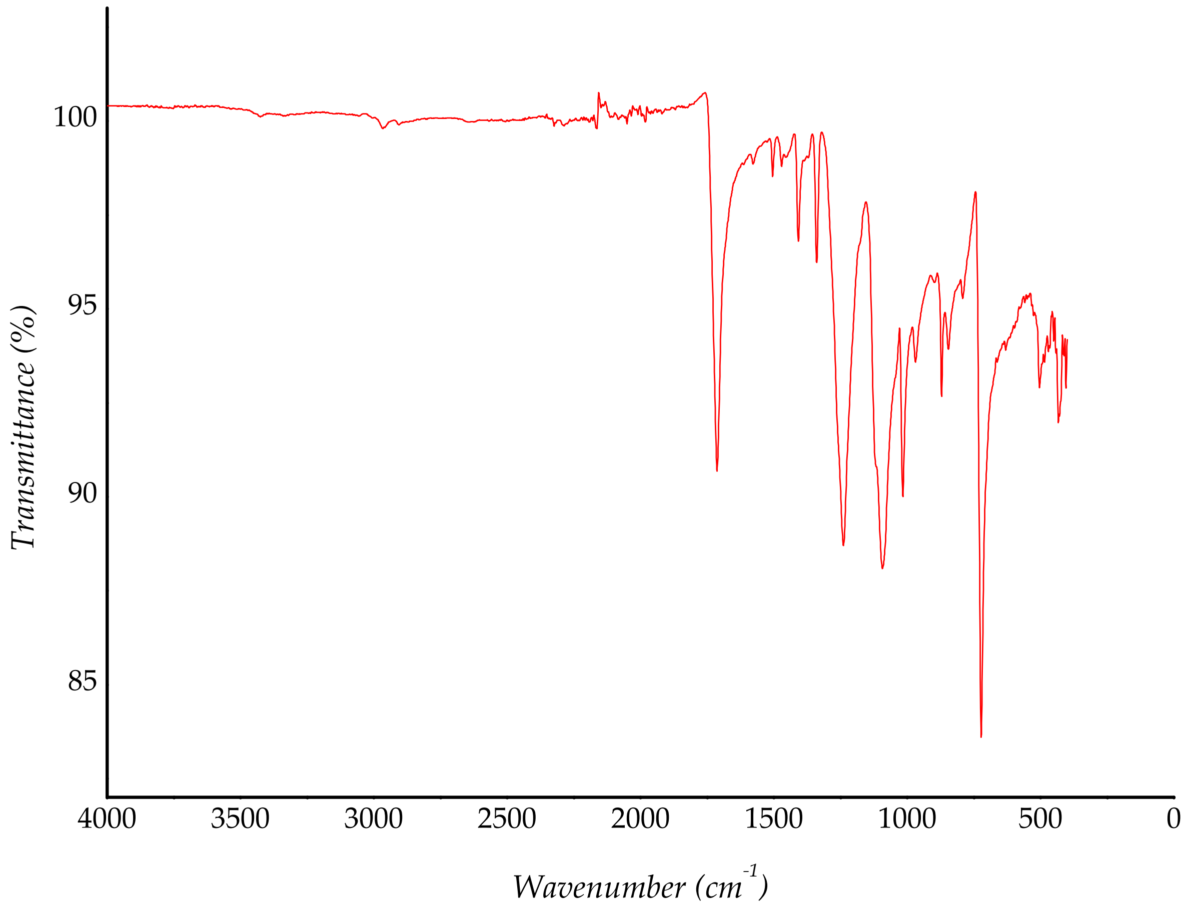

Figure S8. IR spectrum of compound **F3su24**

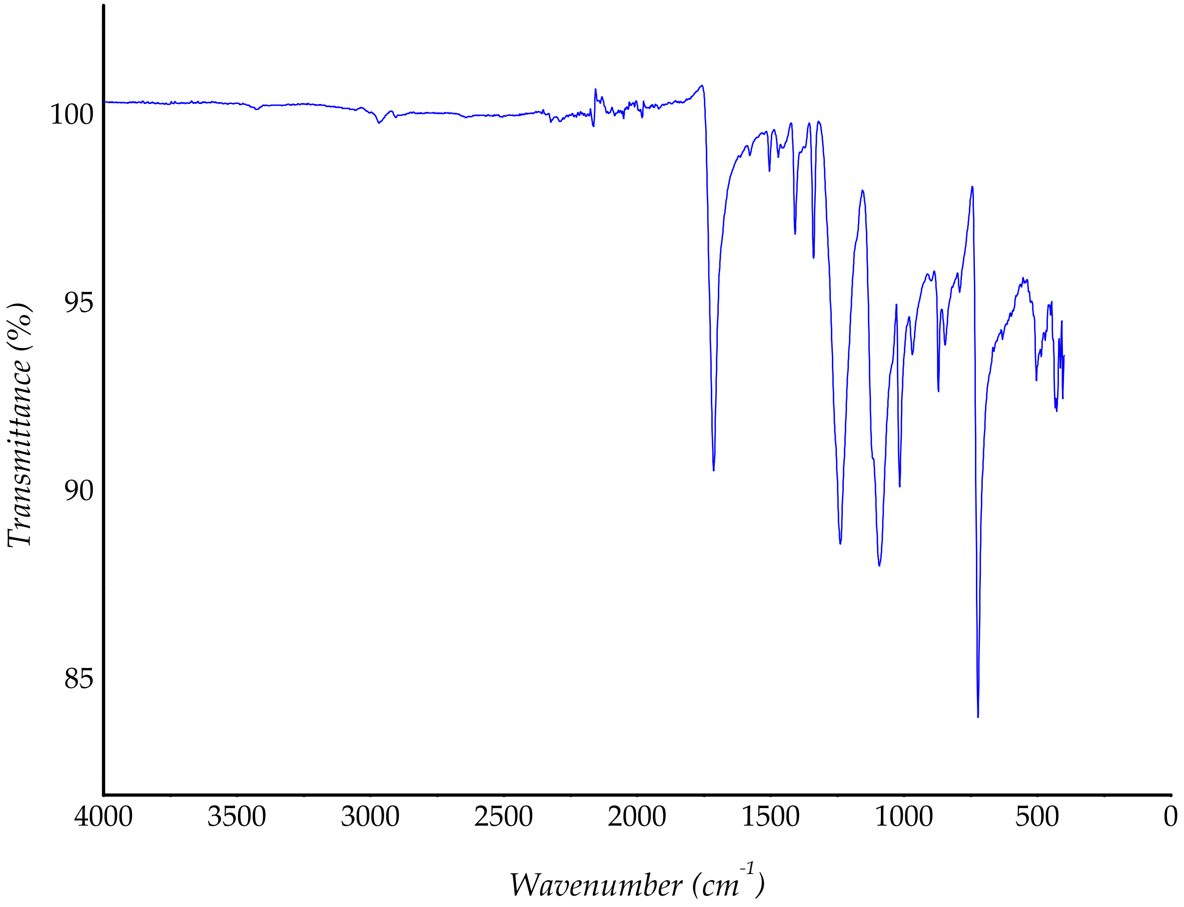

Figure S9. IR spectrum of compound **F3sh24**

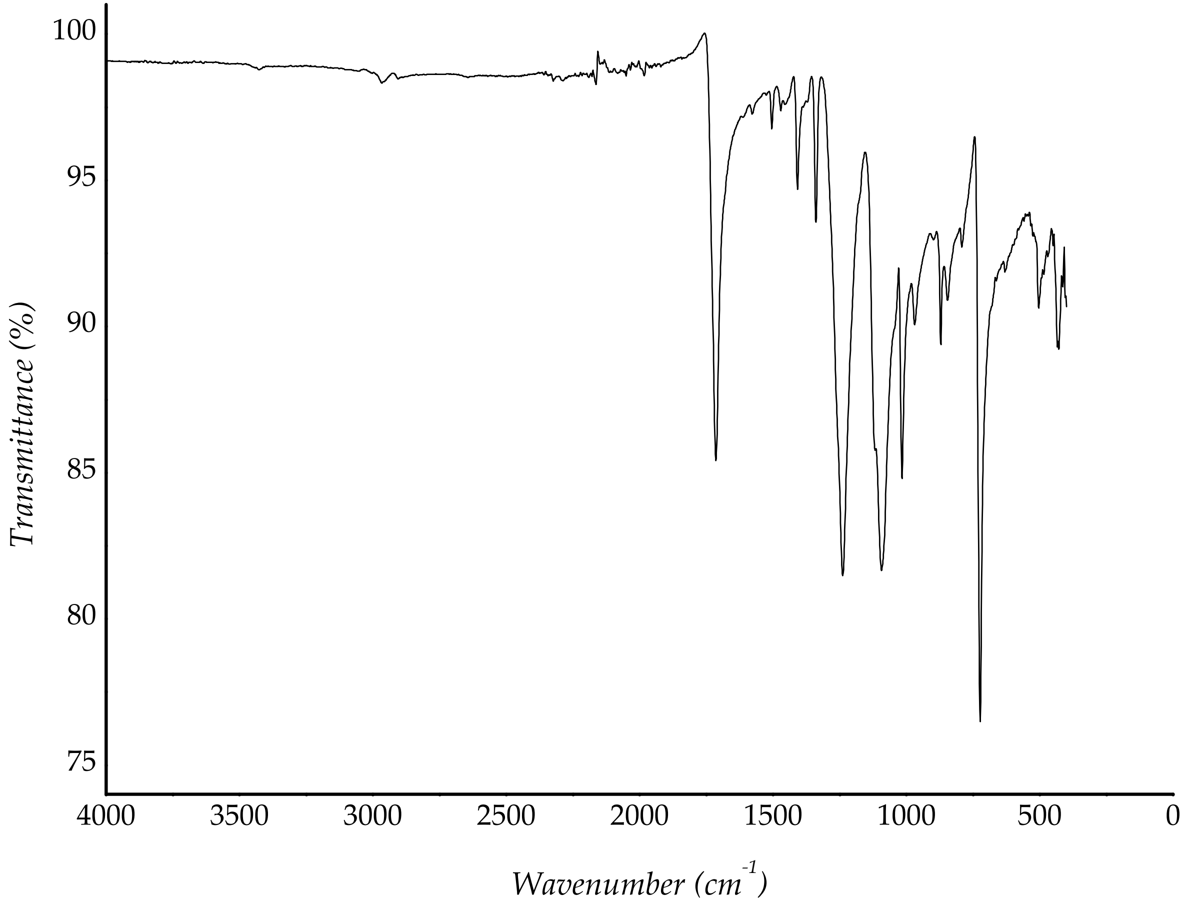

Figure S10. IR spectrum of compound **F4Ø**

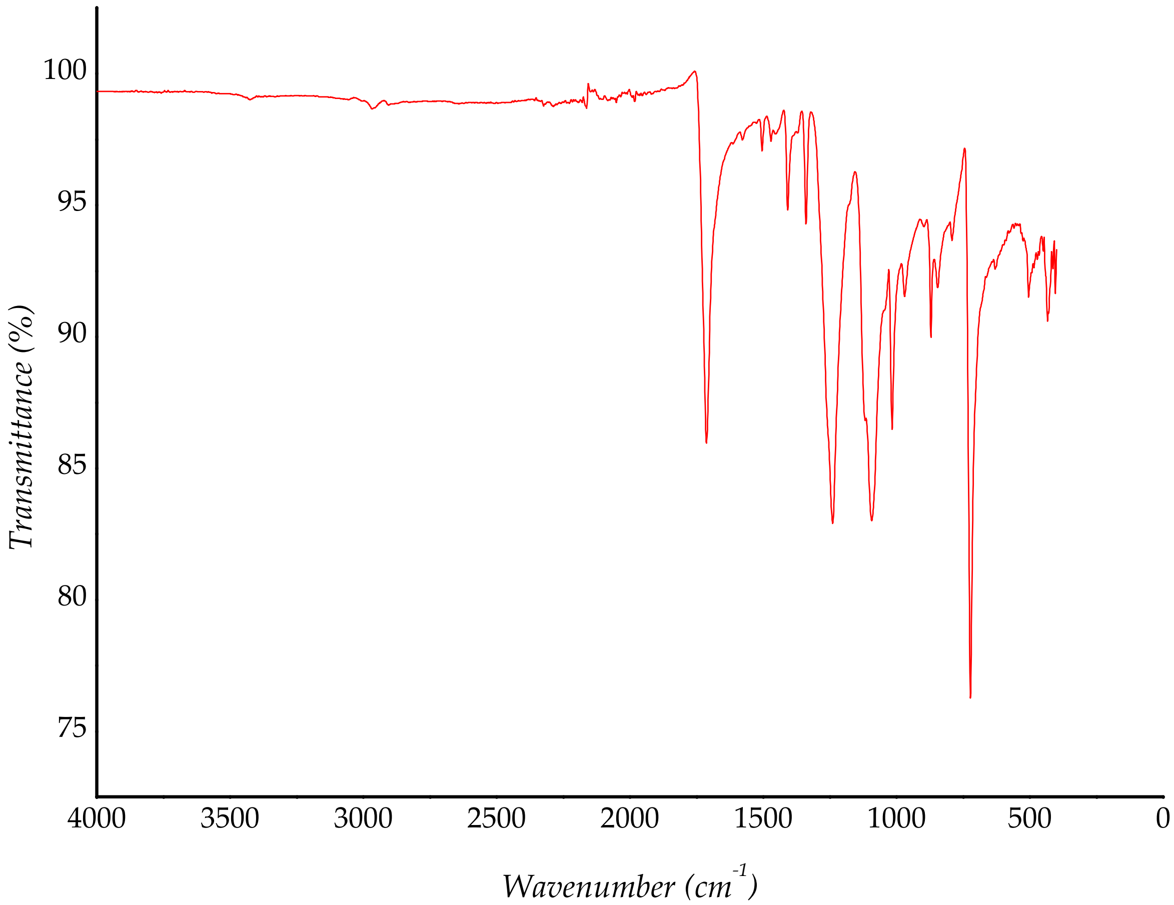

Figure S11. IR spectrum of compound **F4su24**

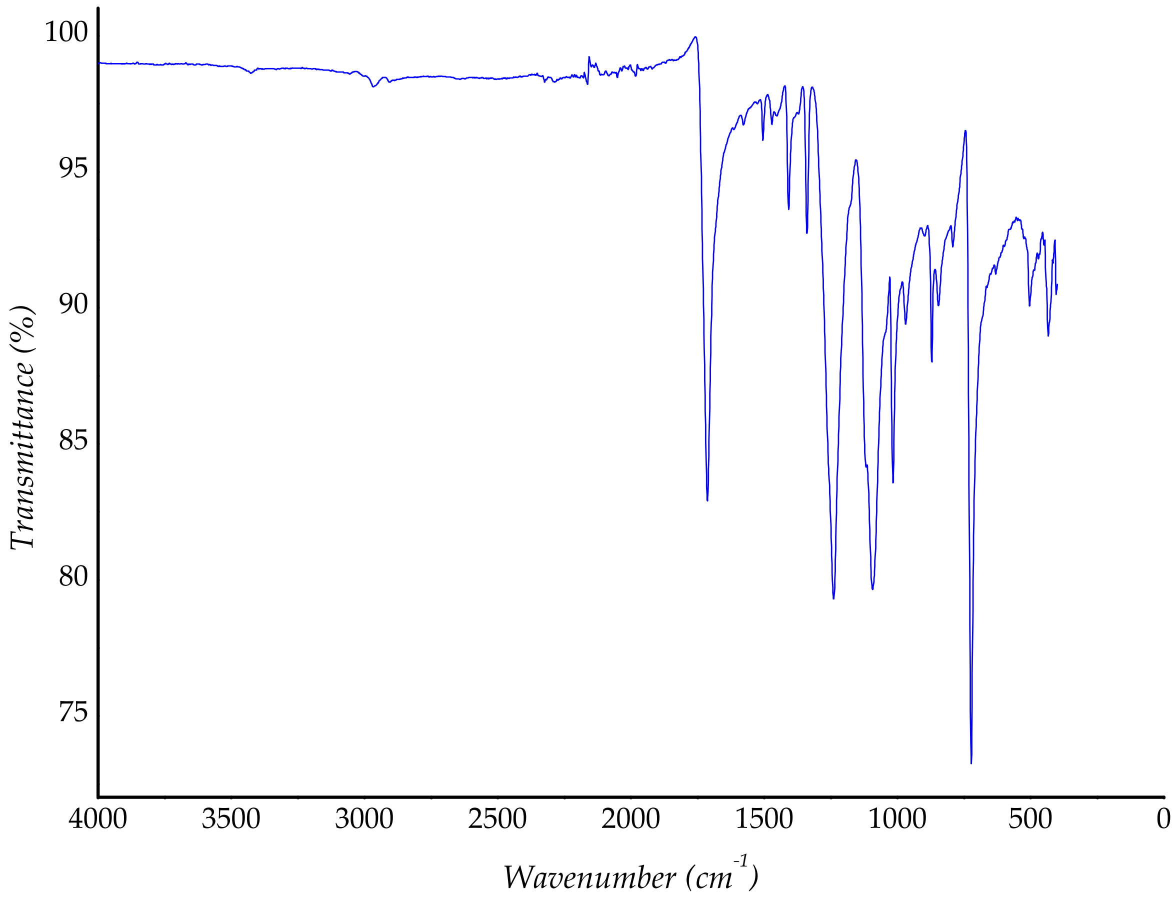

Figure S12. IR spectrum of compound **F4sh24**

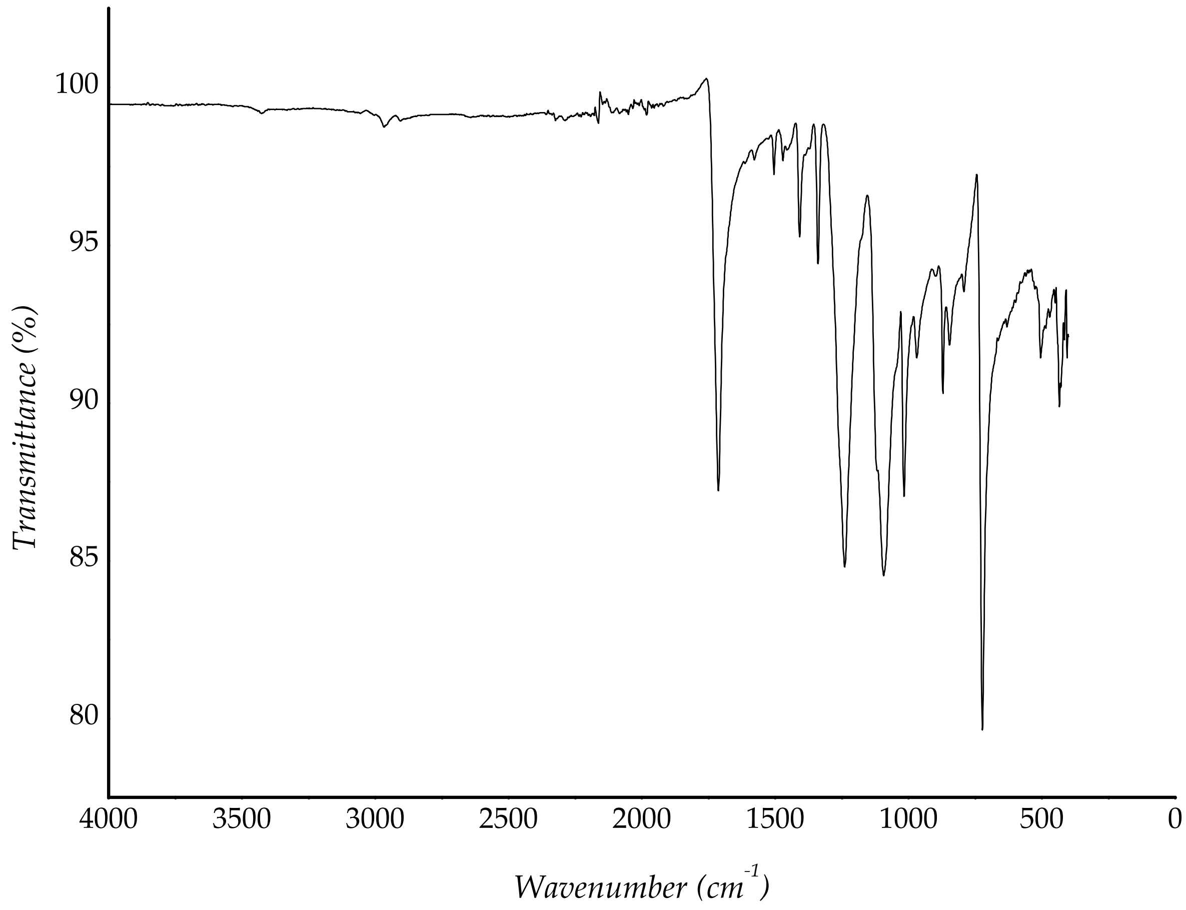

Figure S13. IR spectrum of compound **F5Ø**

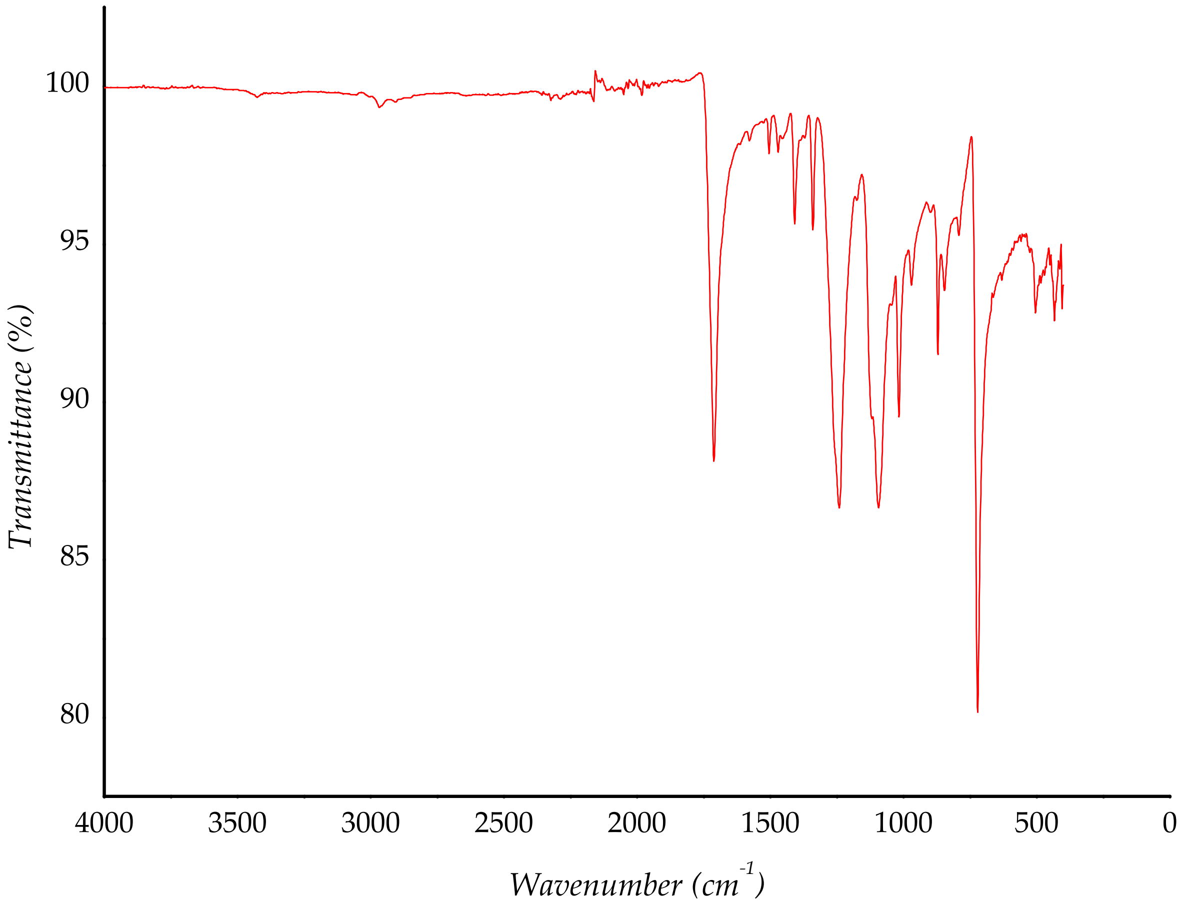

Figure S14. IR spectrum of compound **F5su24**

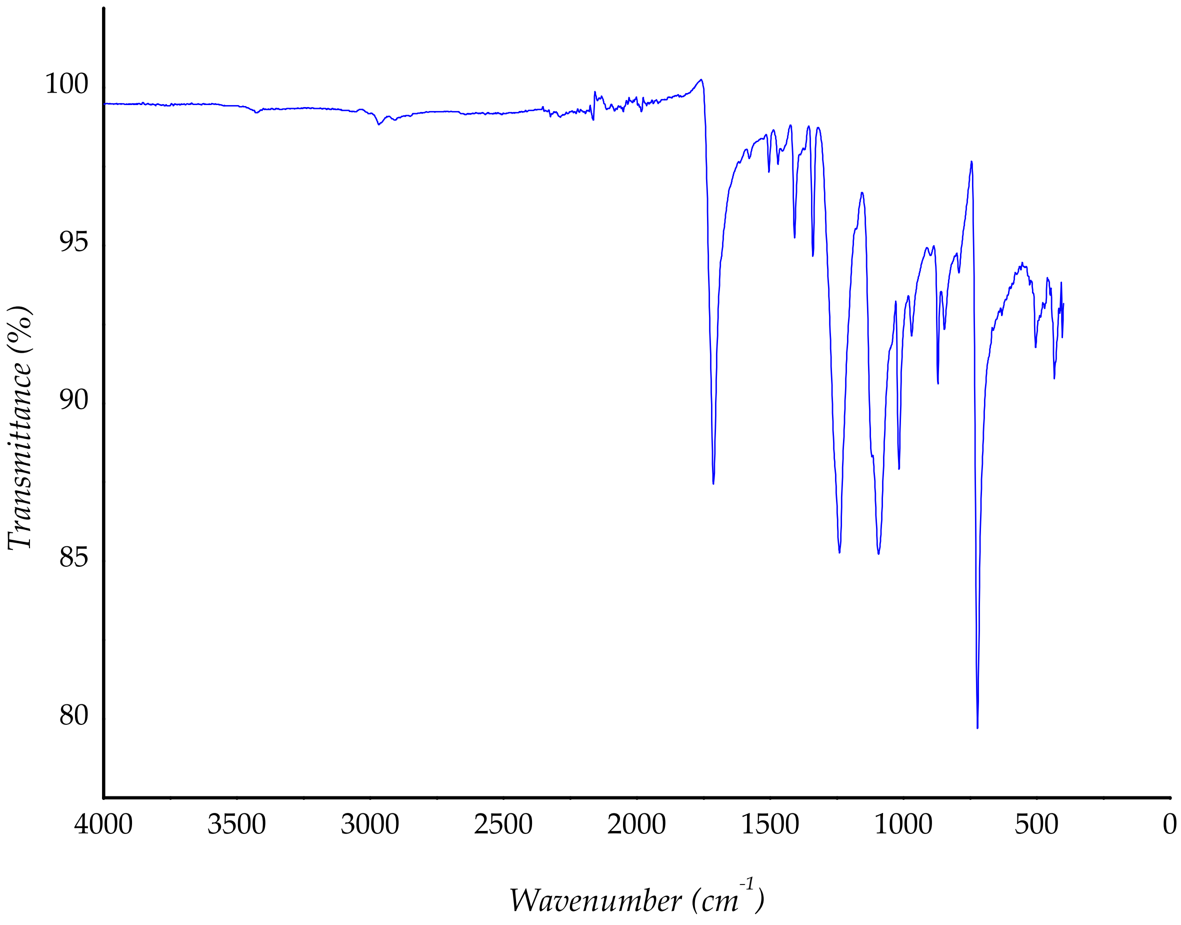

Figure S15. IR spectrum of compound **F5sh24**
